# *SPP1*^*+*^ macrophages in colorectal cancer: Markers of malignancy and promising therapeutic targets

**DOI:** 10.1016/j.gendis.2024.101340

**Published:** 2024-05-30

**Authors:** Zhenyu Xie, Gaozan Zheng, Liaoran Niu, Kunli Du, Ruikai Li, Hanjun Dan, Lili Duan, Hongze Wu, Guangming Ren, Xinyu Dou, Songchen Dai, Fan Feng, Jian Zhang, Jianyong Zheng

**Affiliations:** a Department of Digestive Surgery, Xijing Hospital of Digestive Diseases, Medical University, Xi'an, Shaanxi 710032, China; b Xi'an Medical University, Xi'an, Shaanxi 710021, China; c Department of Surgical Oncology and General Surgery, The First Hospital of China Medical University, Shenyang, Liaoning 110016, China; d Key Laboratory of Precision Diagnosis and Treatment of Gastrointestinal Tumors, Ministry of Education, Shenyang, Liaoning 110016, China; e The State Key Laboratory of Cancer Biology, Department of Biochemistry and Molecular Biology, Air Force Medical University, Xi'an, Shaanxi 710032, China

**Keywords:** Colorectal cancer, Immunotherapy, Single-cell RNA sequencing, Spatial transcriptomics, *SPP1*^+^macrophages

## Abstract

*SPP1^+^* macrophages have been identified as key players in the colorectal cancer (CRC) tumor microenvironment, but their function remains unclear. This study integrated single-cell and spatial transcriptomics with bulk sequencing to investigate the roles and mechanisms of *SPP1*^+^ macrophages in CRC. Our findings revealed a pronounced elevation of *SPP1*^+^ macrophages in CRC, especially within tumor territories. These macrophages served as markers for CRC initiation, progression, metastasis, and potential prognosis. Furthermore, they showed heightened transcriptional activity in genes linked to angiogenesis, epithelial–mesenchymal transition, glycolysis, hypoxia, and immunosuppression. *SPP1* protein amplified CRC cell migration and invasion, potentially mediating cellular crosstalk via the *SPP1-CD44*, *SPP1-PTGER4*, and *SPP1-a4b1* complex axes. Patients with a high proportion of *SPP1*^+^ macrophages could benefit more from immune checkpoint blockade therapy. Interestingly, *CSF1R* expression was significantly enriched in *C1QC*^+^ macrophages versus *SPP1*^+^ macrophages, possibly explaining limited anti-CSF1R monotherapy effects. In conclusion, we propose an *SPP1*^+^ macrophage model in CRC, highlighting such macrophages as a promising therapeutic target due to their malignancy markers.

## Introduction

Colorectal cancer (CRC) is the third most common malignancy and the second leading cause of cancer-related death.^1^ Following the great success of immune checkpoint blockade in various advanced solid tumors, interest in immunotherapy for CRC is increasing.^2^ However, immunotherapy targeting PD-1 (programmed death-1) is only effective in patients with DNA mismatch repair deficiency/high levels of microsatellite instability, which is present in 5% of metastatic CRC cases.^3^, ^4^, ^5^ Therefore, it is necessary to understand the complex mechanisms of cellular and molecular interactions in the CRC tumor microenvironment (TME) and to search for potential targets to develop new immunotherapies.

Macrophages play a crucial role in the TME and are involved in various aspects of tumor immunity.^6^ *In vitro*, M1 (inflammatory/antitumoral) and M2 (anti-inflammatory/protumoral) macrophage polarization systems have been used to classify the activation state of macrophages.^7^ However, *in vivo*, macrophages exhibit a more complex phenotype, which contradicts this simplistic *in vitro* classification.^8^ In the TME, macrophages are often referred to as tumor-associated macrophages, a heterogeneous cell type that contributes to angiogenesis,^9^ extracellular matrix remodeling,^6^ promotes epithelial–mesenchymal transition (EMT) of tumor cells,^10^ and activates immunosuppression,^11^ promoting the progression and metastasis of CRC.^12^ Targeting tumor-associated macrophages via immunotherapies, such as those reducing the number of tumor-associated macrophages in the TME by blocking the *CSF1* (colony-stimulating factor 1)-*CSF1R* (colony-stimulating factor 1 receptor) axis,^13^, ^14^, ^15^ has been applied in the clinic, but their efficacy as monotherapy in malignancies has been minimal.^15^^,^^16^

Single-cell studies have shown that *SPP1*^+^ macrophages play a crucial role in the TME, and are associated with poor prognosis in CRC patients.^17^, ^18^, ^19^, ^20^ *SPP1* (secreted phosphoprotein 1), also known as osteopontin, is overexpressed in various cancer types^21^, ^22^, ^23^ and is implicated in promoting CRC progression and metastasis through EMT and hypoxia pathways.^24^^,^^25^ SPP1 is known to bind to its receptors *CD44* and integrin, regulating several signaling pathways including ERK (extracellular signal-regulated kinase), JNK1 (c-Jun N-terminal kinase 1), and PI3K (phosphoinositide 3-kinase)/Akt (protein kinase B).^26^, ^27^, ^28^, ^29^, ^30^, ^31^ However, the functions and mechanisms of *SPP1*^+^ macrophages remain unknown, and a comprehensive investigation of *SPP1*^+^ macrophages is lacking.

Recent advances in single-cell RNA-sequencing (scRNA-seq) have provided new insights into the complexity of myeloid cells, particularly macrophages, in different tumor types.^17^^,^^19^^,^^32^^,^^33^ Single-cell evidence suggests that *SPP1*^+^ macrophages play a key role in revealing the heterogeneity and functional changes of macrophages during CRC liver metastasis (CRLM).^17^^,^^18^ Using public CRC single-cell datasets, we constructed the largest CRC myeloid cell atlas to date and conducted a systematic investigation of *SPP1*^+^ macrophages. Based on the results, we proposed the *SPP1*^+^ macrophage model, which can explain the clinical characteristics and functional changes of macrophages in CRC and help guide the development of macrophage-targeted immunotherapies.

## Materials and methods

### Material

The public datasets used in this study are from Gene Expression Omnibus (GEO) and The Cancer Genome Atlas (TCGA) and the website that stores the scRNA-seq data of Wu et al. These datasets include two spatial transcriptomics datasets, eight scRNA-seq datasets, and fifteen bulk RNA-seq datasets (Table 1). To ensure the stability and comparability of single-cell data, only scRNA-seq datasets based on the 10x Genomics platform were included in this study; seven CRC datasets (GSE132465, GSE144735, GSE146771, GSE164522, GSE178318, GSE178341, and the dataset of Wu et al) and one hepatocellular carcinoma (HCC) dataset (GSE156625) met this criterion. GSE164522 and the dataset of Wu et al were further defined as CRLM datasets based on tissue sample type, including CRC, normal colorectum (NC), liver metastasis (LM), and peripheral blood mononuclear cell (PBMC) samples. The bulk RNA-seq dataset included high-throughput sequencing data from TCGA-CRC and 14 GEO microarray datasets (GSE6988, GSE14333, GSE17536, GSE20842, GSE20916, GSE39582, GSE41258, GSE44076, GSE44861, GSE68468, GSE83889, GSE87211, GSE106582, and GSE161158). Transcriptome data and clinical information of TCGA-CRC were downloaded from UCSC Xena (http://xena.ucsc.edu/), and single nucleotide variation data (VarScan) were downloaded from the Genomic Data Commons (GDC) portal (https://portal.gdc.cancer.gov/). The transcriptome and clinical information of the GEO dataset were downloaded from the GEO database (https://www.ncbi.nlm.nih.gov/geo/).

**Table 1:** Sources of the ST, scRNA-seq, and bulk RNA-seq datasets.

Deposited data	Platform	Source	Identifier
Human NC ST dataset	10x Genomics	Fawkner-Corbett et al^34^	GEO: GSE158328
Human CRC ST dataset	10x Genomics	Wu et al^18^	http://www.cancerdiversity.asia/scCRLM
Human LM ST dataset	10x Genomics	Wu et al^18^	http://www.cancerdiversity.asia/scCRLM
Human CRC scRNA-seq dataset	10x Genomics	Lee et al^35^	GEO: GSE132465
Human CRC scRNA-seq dataset	10x Genomics	Lee et al^35^	GEO: GSE144735
Human CRC scRNA-seq dataset	10x Genomics	Zhang et al.^19^	GEO: GSE146771
Human CRC scRNA-seq dataset	10x Genomics	Che et al.^36^	GEO: GSE178318
Human CRC scRNA-seq dataset	10x Genomics	Pelka et al^37^	GEO: GSE178341
Human CRLM scRNA-seq dataset	10x Genomics	Liu et al^17^	GEO: GSE164522
Human CRLM scRNA-seq dataset	10x Genomics	Wu et al^18^	http://www.cancerdiversity.asia/scCRLM
Human HCC scRNA-seq dataset	10x Genomics	Sharma et al^38^	GEO: GSE156625
Human CRC bulk RNA-seq dataset	Illumina Hiseq	–	TCGA
Human CRC bulk RNA-seq dataset	GPL4811	Ki et al^39^	GEO: GSE6988
Human CRC bulk RNA-seq dataset	GPL570	Jorissen et al^40^	GEO: GSE14333
Human CRC bulk RNA-seq dataset	GPL570	Smith et al^41^	GEO: GSE17536
Human CRC bulk RNA-seq dataset	GPL4133	Gaedcke et al^42^	GEO: GSE20842
Human CRC bulk RNA-seq dataset	GPL570	Skrzypczak et al^43^	GEO: GSE20916
Human CRC bulk RNA-seq dataset	GPL570	Marisa et al^44^	GEO: GSE39582
Human CRC bulk RNA-seq dataset	GPL96	Sheffer et al^45^	GEO: GSE41258
Human CRC bulk RNA-seq dataset	GPL13667	Sole et al^46^	GEO: GSE44076
Human CRC bulk RNA-seq dataset	GPL3921	Ryan et al^47^	GEO: GSE44861
Human CRC bulk RNA-seq dataset	GPL96	Sheffer et al^45^	GEO: GSE68468
Human CRC bulk RNA-seq dataset	GPL10558	Kwon et al^48^	GEO: GSE83889
Human CRC bulk RNA-seq dataset	GPL13497	Hu et al^49^	GEO: GSE87211
Human CRC bulk RNA-seq dataset	GPL10558	–	GEO: GSE106582
Human CRC bulk RNA-seq dataset	GPL570	Szeglin et al^50^	GEO: GSE161158

Note: ST, spatial transcriptomics; HCC, hepatocellular carcinoma; scRNA-seq, single-cell RNA-sequencing; RNA-seq, RNA-sequencing; CRC, colorectal cancer; NC, normal colorectum (adjacent colorectum); LM, liver metastases; CRLM, CRC liver metastasis.

### Basic analysis workflow for scRNA-seq data

The R package Seurat (version 4.1.0) converted raw unique molecular identifier count matrices to Seurat objects and then filtered the data according to the following criteria: i) filtering genes expressed in at least 10 cells; ii) filtering cells with unique feature counts over 6,000 or less than 500; and iii) filtering cells with >20% mitochondrial counts. After quality control, “*LogNormalize*” was used to normalize the expression of all cells with a scale factor of 10,000. The top 2000 highly variable genes were identified based on the mean and dispersion. During scaling of the highly variable genes, the “*var.to.regress*” option was used to regress the percent mitochondrial content. The results were obtained by principal component analysis for linear dimensionality reduction. We used the “*FindClusters*” function on 50 principal components with a resolution of 0.8 for preliminary clustering and annotation. Afterward, the visualization of cell clustering was completed by the nonlinear dimensionality reduction UMAP method.^51^ Finally, the main cell types were identified and annotated based on canonical marker genes. Subsequent annotation of myeloid cell subsets followed the same workflow described above for major cell types.

### Annotation of major cell types and myeloid cell subsets

The primary methodology for categorizing clusters into specific major cell types and myeloid cell subsets in this research was based on the expression level of essential marker genes within these clusters.

Myeloid cells were defined based on the similarity of cell clusters rather than strict adherence to biological definitions. As a result, in the cluster annotation of major cell types, real myeloid cells such as neutrophils and mast cells were named independently, while plasmacytoid dendritic cells of lymphoid origin,^52^ Kupffer cells derived from embryonic yolk sac,^53^ and even doublets were all classified as myeloid cells.

Given that CRC macrophages were the focus of this study, the major monocyte/macrophage subpopulation in PBMCs of the CRLM dataset was broadly defined as monocytes. In datasets without PBMC samples, we did not define the monocyte subset. Due to the high similarity in the transcriptome characteristics of monocytes and Macro-*FCN1*, potential monocytes in these datasets were usually classified into the Macro-*FCN1* subset. Similarly, in datasets without NL samples, we did not define a subset of Kupffer cells.

Macro-*C1QC*, Macro-*FCN1*, Macro-*MKI67*, Macro-*SPP1*, monocytes, and Kupffer cells in this study were collectively referred to as monocytes/macrophages; macrophages included Macro-*C1QC*, Macro-*FCN1*, Macro-*MKI67*, and Macro-*SPP1* subsets.

### Data integration and batch correction

To construct the large sample data of CRC myeloid cells, CRC myeloid cell data from five independent datasets (GSE132465, GSE144735, GSE146771, GSE178318, and GSE178341) were combined into CRC-Mix data. After correction for batch effects using the harmony algorithm,^54^ the CRC-Mix data were reclustered into groups and annotated.

In addition, to compare the differences in *SPP1*^+^ macrophage proportions in CRLM and HCC, myeloid cells of GSE156625 and GSE164522 were integrated in the same way, subjected to batch effect correction by the harmony algorithm, reclustered into groups, and annotated.

### Expression difference analysis and representative genes

The “*Findallmarkers*” function in Seurat was used to identify marker genes for each cluster or subset. In addition, to explore the functional changes in monocytes/macrophages during CRLM, we also used the “*Findmarkers*” function to compare the monocyte/macrophage transcriptomes of NC and CRC, as well as CRC and LM. The parameters assert = “RNA” and slot = “counts” allowed for the inclusion of genes with low variability in the comparison. Genes with significant expression differences (*P* < 0.05) in the comparison between groups were defined as differentially expressed genes (DEGs). DEGs with the same trend in multiple datasets were defined as common DEGs, and these genes were used for subsequent functional enrichment analysis.

In brief, for myeloid cell subsets in the CRC-Mix dataset, genes that met these criteria were considered representative genes: i) adjusted *P* value < 0.01; ii) log fold-change of the average expression >0.5; iii) pct.1 (percentage of gene expression detected in the first group) > 0.25; iv) the above three-step filtering process was also performed on the myeloid cell subsets defined before harmony correction, and the intersection genes were selected. These genes were further checked manually to ensure their expression specificity on the corresponding subsets.

### Tissue distribution of myeloid cell subsets

To understand the distribution preference of each myeloid cell subtype in different tissues, we evaluated the enrichment of each myeloid cell subtype using odds ratios in seven CRC single-cell datasets. The odds ratio >1 indicates the relative enrichment of the cell subtype in a specific tissue.

### Monocyte/macrophage developmental trajectory in CRLM

To construct the monocyte/macrophage developmental trajectory of CRLM, we used the “Monocle” package (version 2.22.0)^55^ to align monocytes/macrophages in pseudotime order. The *DDRTree* method implemented with the “*reduceDimension*” function of Monocle 2 was used for dimensionality reduction and construction of pseudo-temporal order.

### Cell–cell interaction analysis

We used Python-based (version 3.7) CellphoneDB^56^ to evaluate the interactions of macrophages with other cells. The putative ligands and receptors were determined based on whether they were expressed in each cell. To efficiently assess the closeness of cell interactions based on the number of receptor–ligand pairs, we randomly sampled 1000 cells per population from the Macro-*C1QC*, Macro-*FCN1*, Macro-*MKI67*, Macro-*SPP1*, and major cell types in the GSE178318 dataset.

### Definition of phenotype scores

To understand the characteristics between different macrophage subsets, scores for different phenotypes were obtained by the “*AddModuleScore*” function in the “*Seurat*” package. These scores were defined by the mean expression of phenotype-related genes.

The related genes for M1 and M2 macrophages were defined as the M1 score and M2 score, respectively^57^ (Table S1). Similarly, the inflammatory score, anti-inflammatory score,^35^ glycolysis score,^58^ hypoxia score,^58^ EMT score,^59^ proliferation score,^60^ angiogenesis score,^19^ phagocytosis score,^19^ and MHC-II (major histocompatibility complex class II) score were defined based on their respective sets of genes (Table S1).

### Immune infiltration and macrophage markers

The immune score calculated by the ESTIMATE algorithm was used to assess the overall immune infiltration of each sample.^61^ The immune cell marker *CD45* was also used to assess the level of immune cell infiltration in tissues. CIBERSORT, a deconvolution algorithm based on transcriptional profiles, was used to calculate the proportions of 22 immune cells, including M0, M1, and M2 macrophages, in each sample.^62^ To understand the clinical features of *SPP1* as a specific macrophage marker in CRC, some classic macrophage markers were selected for comparison; these included the pan-macrophage marker *CD68*, M1 macrophage markers *CD86* and *iNOS* (inducible nitric oxide synthase), and M2 macrophage markers *CD163* and *CD206*.^63^^,^^64^ In addition, the web server TIMER (http://timer.cistrome.org/) was used to evaluate the correlation of *SPP1* with the six immune cells and macrophage markers mentioned above.^65^

### Total RNA extraction and quantitative reverse transcription PCR

Fifty pairs of CRC samples and paratumor tissues were obtained from CRC patients who underwent surgery at Xijing Hospital. Total RNA was extracted from the tissues using Trizol reagent (Invitrogen, Waltham, MA, United States), and cDNA was synthesized by reverse transcription using the PrimeScript RT Reagent Kit (TaKaRa, Tokyo, Japan). Quantitative real-time PCR was performed to confirm the expression levels of SPP1, using the SYBR Premix Ex Taq II Kit (TaKaRa, Tokyo, Japan). *GAPDH* was used as an internal control for normalization. The relative mRNA expression was calculated using the 2^−ΔΔCt^ method. The primer sequences used were as follows: *SPP1* forward: 5′-GAAGTTTCGCAGACCTGACAT-3′; *SPP1* reverse: 5′-GTATGCACCATTCAACTCCTCG-3′; *GAPDH* forward: 5′-GACAGTCAGCCGCATCTTCT-3′; *GAPDH* reverse: 5′-GCGCCCAATACGACCAAATC-3′.

### Spatial transcriptomic analysis

The “*SCTransform*” function of Seurat was used to standardize the spatial transcriptomic data of NC, CRC, and LM samples. “*RunPCA*” and “*RunUMAP*” were used for dimensionality reduction and clustering (principal components = 15, resolution = 0.8). After merging similar clusters, we identified spatial transcriptomics-defined regions in NC, CRC, and LM samples. The “*FindAllMarkers*” function was used to analyze the specific genes of each region in the spatial transcriptome (adjusted *P*-value <0.01, |log_2_ fold change| > 0.4). Subsequently, we assessed the distribution of immune cells in spatial transcriptomic regions of CRC and LM samples using multimodal intersection analysis,^66^ which determined the cell type enrichment degrees by performing a hypergeometric test on the overlap between the cell type-specific genes of the scRNA-seq data and the region-specific genes of the spatial transcriptomics data (the scRNA-seq and spatial transcriptomics data of CRC and LM samples were both from the dataset of Wu et al). To explore the functional characteristics of tumor regions, region-specific genes in CRC and LM samples were further used for functional enrichment analysis. In addition, we used the “*AddModuleScore*” function of Seurat to evaluate the spatial distribution of glycolysis and hypoxia signatures.

### Immunofluorescence staining

Human tissue specimens were obtained from Xijing Hospital under the approval of the Institutional Review Board. The CRC-paired specimens were collected within 30 min after the tumor resection and fixed in paraformaldehyde for 48 h. Dehydration and embedding in paraffin were carried out using standard procedures. To inhibit endogenous peroxidase activity, the specimens were treated with 3% H_2_O_2_ for 25 min. Following preincubation with 10% normal goat serum for blocking nonspecific binding sites for 30 min, the tissue sections were incubated at 4 °C with primary antibodies in a humidified chamber overnight. The primary antibodies used for validating *SPP1*^+^ macrophages were as follows: mouse anti-human *CD68* (Proteintech, Cat# 66231-2-Ig, 1:2000) and mouse anti-human *SPP1* (Santa Cruz Biotechnology, Cat# sc-21742, 1:1000). After thorough washing, the sections were mounted with an anti-fading reagent and coverslips were applied. Fluorescence microscopy (NIKON ECLIPSE C1) was used to visualize the images, and subsequent analysis was performed using CaseViewer software.

### Tumor mutation burden

Simple nucleotide variation data from TCGA-CRC were used to calculate tumor mutation burden, which was defined as the number of mutations per megabase. The visualization of the detailed gene mutation status of the high and low *SPP1/CD68* groups was implemented in the R package “maftools”.^67^

### Prediction of immunotherapy response

We used the SubMap algorithm to predict the clinical response to anti-PD-1 and anti-CTLA4 (cytotoxic T-lymphocyte associated protein 4) immunotherapy. SubMap was used to compare the similarity of different expression profiles. This feature can reflect the response to treatment.^68^ The expression profiles and associated annotation data of 47 melanoma patients used to define high- and low-risk groups were obtained from the Supplementary Materials of Lu et al.^69^ In addition, samples in GSE39582 were divided into high and low *SPP1* groups based on the median *SPP1* expression.

### Survival analysis

Overall survival and progression-free interval were regarded as the outcome events in the datasets (overall survival in GSE17536, GSE39582, and GSE41258; overall survival/progression-free interval in TCGA). Survival analysis was performed based on the Kaplan–Meier algorithm. The log-rank test was used to calculate *P*-values between groups. Univariate Cox models were constructed by Cox proportional hazards regression. These analyses were implemented with “*survival*” and “*survminer*” in R.

### Functional and pathway enrichment analysis

Functional and pathway enrichment analysis was conducted using four different gene sets including Gene Ontology (GO) Biological Process (BP), Kyoto Encyclopedia of Genes and Genomes (KEGG), WikiPathways, and Hallmark gene sets.^70^

Metascape (http://metascape.org/), a portal for gene function annotation analysis, was used for enrichment analysis of DEGs in this study (GO BP, KEGG, WikiPathways, and Hallmark gene sets).^71^ Gene Set Variation Analysis (GSVA) implemented in the “GSVA” package (version 1.44.2) was used for gene set enrichment analysis of different macrophage subsets (Hallmark gene sets).^72^ LinkedOmics (http://www.linkedomics.org/) is an online source portal for analyzing multiomics data of 32 cancers in TCGA.^73^ In this study, LinkedOmics was used to perform Gene Set Enrichment Analysis (GSEA) of *SPP1* expression profiles in TCGA-CRC (GO BP). In addition, GSEA software (version 4.2.3) (https://www.gsea-msigdb.org/gsea/) was used to explore differences in functional enrichment between the high (H-*SPP1*/*CD68*) and low (L-*SPP1*/*CD68*) groups (Hallmark gene sets, permutations = 1000).^74^

### Statistical analysis

R (version 4.1.3), GraphPad Prism (version 9), Excel, SPSS (version 26), and Python (version 3.7) were used for statistical analysis. The Mann–Whitney U test was used to compare the differences between two groups, and the Wilcoxon test was used for comparing paired samples. The Pearson or Spearman method was used for the correlation test. The log-rank method was used to calculate the *P-*values in survival analysis between groups. A chi-square test was used to compare clinical parameters between the high and low *SPP1/CD68* groups. Data in bar plots of cell proportions for single-cell datasets were presented as mean ± standard error of the mean (small sample size), while mean ± standard deviation was used for the remaining data. *P*-values <0.05 were considered statistically significant.

## Results

### Identification of myeloid cell subsets in CRC

**Figure 1:**
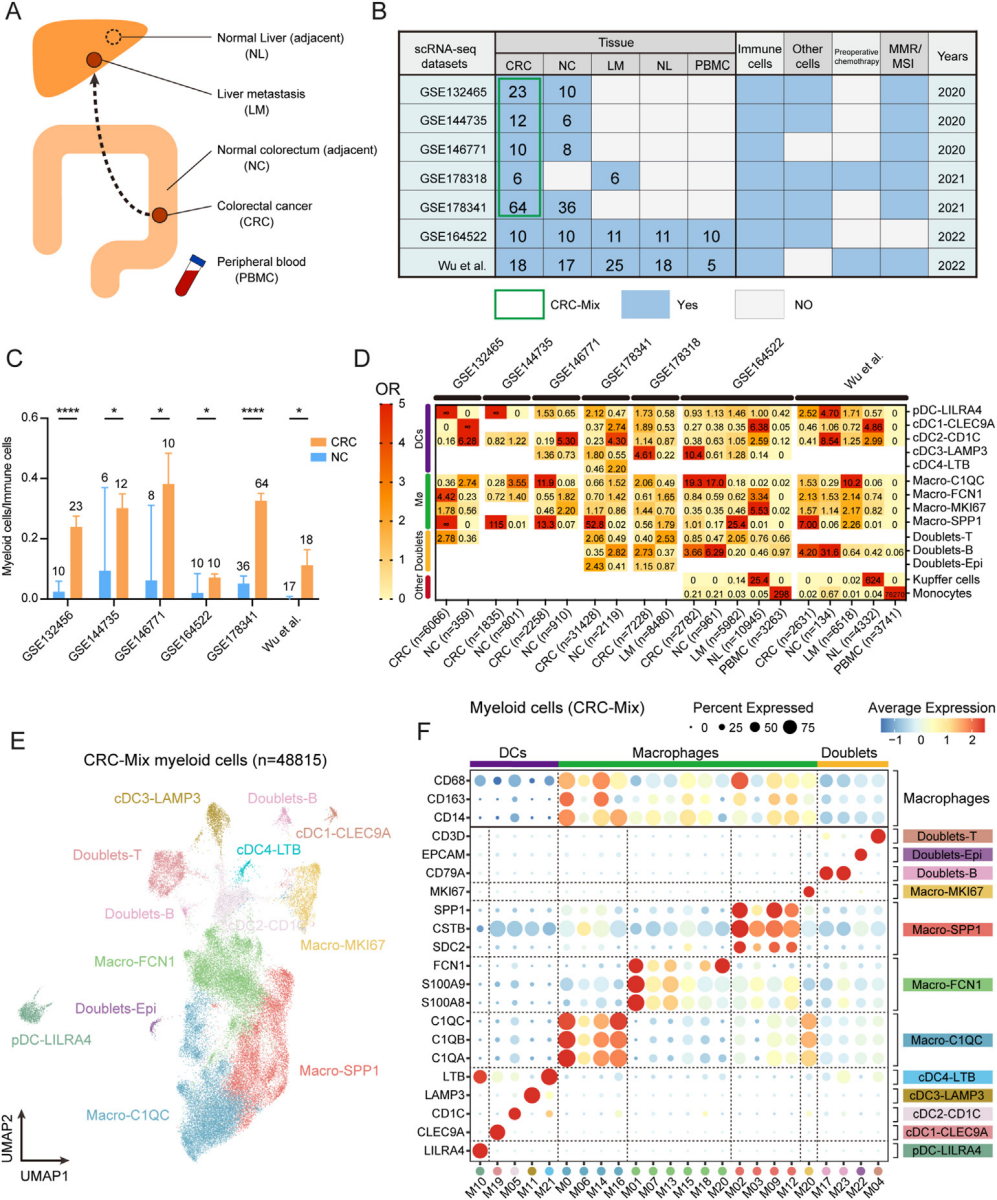
Identification of myeloid cell subsets in CRC. **(A)** Tissue types of CRLM, including CRC, NC, LM, and NL samples and PBMCs. NC and NL samples include both adjacent and healthy tissue. **(B)** Summary of the seven CRC single-cell datasets in this study. The green box indicates the sample origin of the CRC-Mix dataset. Numbers indicate sample size. Other cells refer to non-immune cells including malignant cells and stromal cells. **(C)** Bar plots of the comparison of the proportion of myeloid cells to immune cells between the NC and CRC groups in six CRC scRNA-seq datasets. The sample size is shown above the bar. ∗*P* < 0.05, ∗∗∗∗*P* < 0.0001; mean ± standard error of the mean; Mann–Whitney U test. **(D)** Tissue distribution preference of each myeloid subset estimated by odds ratios (ORs). **(E)** UMAP plot of 12 myeloid subsets from the CRC-Mix dataset. **(F)** Dot plot of the scaled average expression and percentage of expression for key marker genes per myeloid cluster in the CRC-Mix dataset. CRC, colorectal cancer; CRLM, colorectal cancer liver metastases; NC, normal colorectum (adjacent colorectum); LM, liver metastases; NL, normal liver (adjacent); PBMCs, peripheral blood mononuclear cells; MMR, mismatch repair; MSI, microsatellite instability.

**Figure S1:**
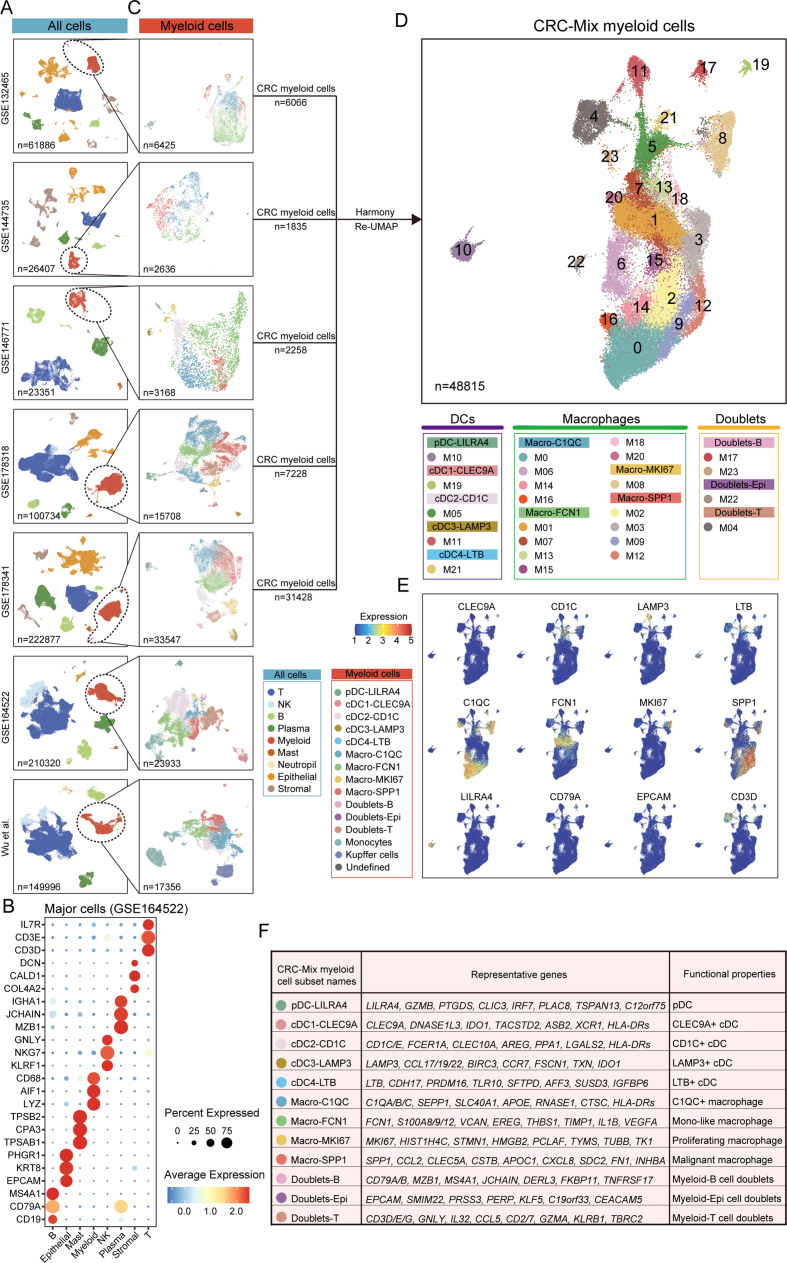
Construction of the CRC-Mix cohort and annotation of myeloid cells. **(A)** UMAP plots of major cell types identified in the seven CRC datasets. Each dot represents a cell colored by cell type. **(B)** Dot plots of marker genes for each major cell type in the GSE164522 dataset. **(C)** UMAP plots of myeloid cell subsets identified in the seven CRC datasets. **(D)** UMAP plot of myeloid cell clusters in CRC-Mix grouped after harmony correction. These cell clusters were further classified into specific myeloid cell subsets (bottom). **(E)** UMAP plots of the marker gene expression of myeloid cell subsets. **(F)** Representative genes and functional properties of myeloid subsets of the CRC-Mix dataset. CRC, colorectal cancer.

This study involved analysis of tissue types including CRC, NC, LM, NL, and PBMCs (Fig. 1A). CRC single-cell data were obtained from seven publicly available datasets (GSE132465, GSE144735, GSE146771, GSE164522, GSE178318, GSE178341, and the dataset of Wu et al) (Fig. 1B). Nine major cell types were identified in these datasets, including T cells (*CD3D*), natural killer cells (*KLRF1*), B cells (*MS4A1*), plasma cells (*MZB1*), myeloid cells (*LYZ*), mast cells (*TPSAB1*), neutrophils (*FCGR3B*), epithelial cells (*EPCAM*), and stromal cells (*DCN*) (Fig. S1A, B). Notably, the proportion of myeloid cells among all immune cells was significantly higher (6/6) in CRC samples compared with NC samples (Fig. 1C and Table S2), suggesting an important role for myeloid cells in the CRC tumor immune microenvironment.

We defined 14 distinct myeloid cell subsets, consisting of one plasmacytoid dendritic cell subset (pDC-*LILRA4*, defined by *LILRA4* expression), four conventional DC subsets (cDC1-*CLEC9A*, cDC2-*CD1C*, cDC3-*LAMP3*, and cDC4-*LTB*), four macrophage subsets (Macro-*C1QC*, Macro-*FCN1*, Macro-*MKI67*, and Macro-*SPP1*), and three doublet subsets (Doublets-B, Doublets-Epi, and Doublets-T), as well as the monocyte subset (*CD14*) and Kupffer cells (*MARCO*) (Fig. S1C; Table S2). We then examined the distribution of these myeloid cell subsets in each of the seven cohorts using odds ratios (Fig. 1D). Our analysis revealed that the Macro-*SPP1* subset was enriched in CRC samples.

To create a more accurate model of the CRC myeloid cell lineage, we integrated CRC myeloid cells from five datasets (GSE132465, GSE144735, GSE146771, GSE178318, and GSE178341) into a large cohort (*n* = 48,815) called CRC-Mix (Fig. S1D), which was then reclustered and annotated after batch effect correction. This resulted in the identification of 12 myeloid cell subsets in CRC tissue (Fig. 1E, F; Fig. S1E), along with a summary table of representative genes for aiding other researchers in identifying these subsets (Fig. S1F). Further details on DEGs between subsets can be found in Table S3.

### Changes in monocytes/macrophages during CRLM

**Figure 2:**
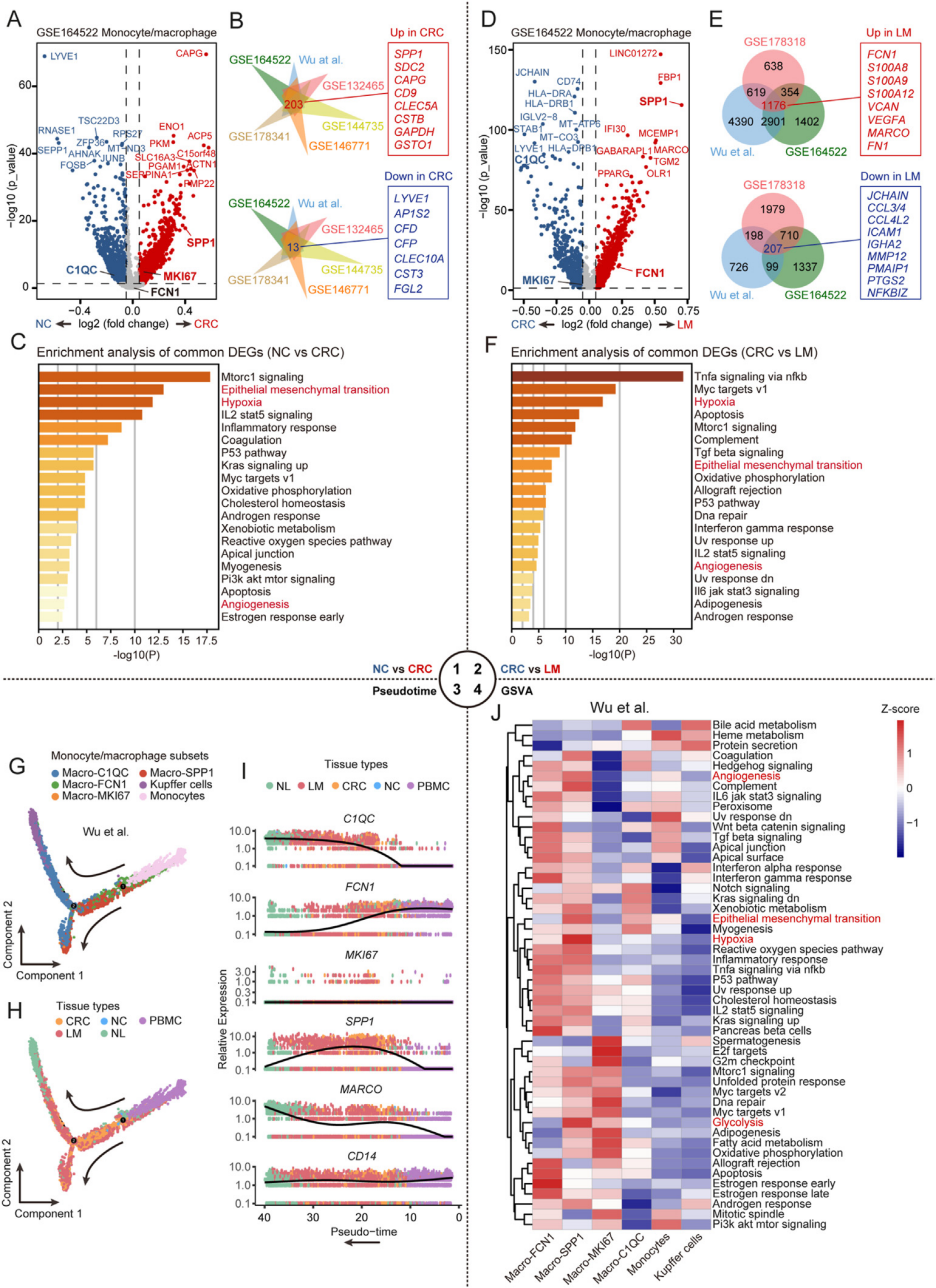
Changes in monocytes/macrophages during CRLM. **(A)** Volcano plot of DEGs in monocytes/macrophages between NC and CRC samples in the GSE164552 dataset. **(B)** Venn diagrams of common DEGs up- or down-regulated across the six scRNA-seq datasets (NC *vs*. CRC). **(C)** Metascape bar graph of functional enrichment analysis results for common DEGs (NC *vs*. CRC). The top 20 enriched hallmark terms are displayed. **(D)** Volcano plot of DEGs in monocytes/macrophages between CRC and LM samples in the GSE164552 dataset. **(E)** Venn diagrams of common DEGs up- or down-regulated across the three scRNA-seq datasets (CRC *vs*. LM). **(F)** Metascape bar graph of functional enrichment analysis results for common DEGs (CRC *vs*. LM). The top 20 enriched hallmark terms are displayed. **(G–I)** Trajectory analysis of monocytes/macrophages (Wu et al), including color-coded subsets (G), tissue types (H), and gene expression levels (I). **(J)** Heatmap of GSVA (Gene Set Variation Analysis) scores of 50 hallmark pathways for different monocyte/macrophage subsets (Wu et al). CRC, colorectal cancer; CRLM, colorectal cancer liver metastases; NC, normal colorectum (adjacent colorectum); DEGs, differentially expressed genes; LM, liver metastases.

We used single-cell sequencing to examine changes in the transcriptional profile and functional pathways of monocytes/macrophages during CRLM. We identified DEGs of monocytes/macrophages between NC and CRC samples (GSE164522) and in six CRC single-cell datasets (NC *vs*. CRC) (Fig. 2A, B and Table S4). Enrichment analysis of the common DEGs (*n* = 216) revealed angiogenesis-, EMT-, and hypoxia-related pathway changes in monocytes/macrophages during CRC occurrence (Fig. 2C). Similarly, we identified DEGs of monocytes/macrophages between CRC and LM samples (GSE164522) and in three CRC single-cell datasets (CRC *vs*. LM) (Fig. 2D, E and Table S5), with enrichment analysis of the common DEGs (*n* = 1383) revealing angiogenesis-, EMT-, and hypoxia-related pathway changes in monocytes/macrophages during liver metastasis of CRC (Fig. 2F).

**Figure S2:**
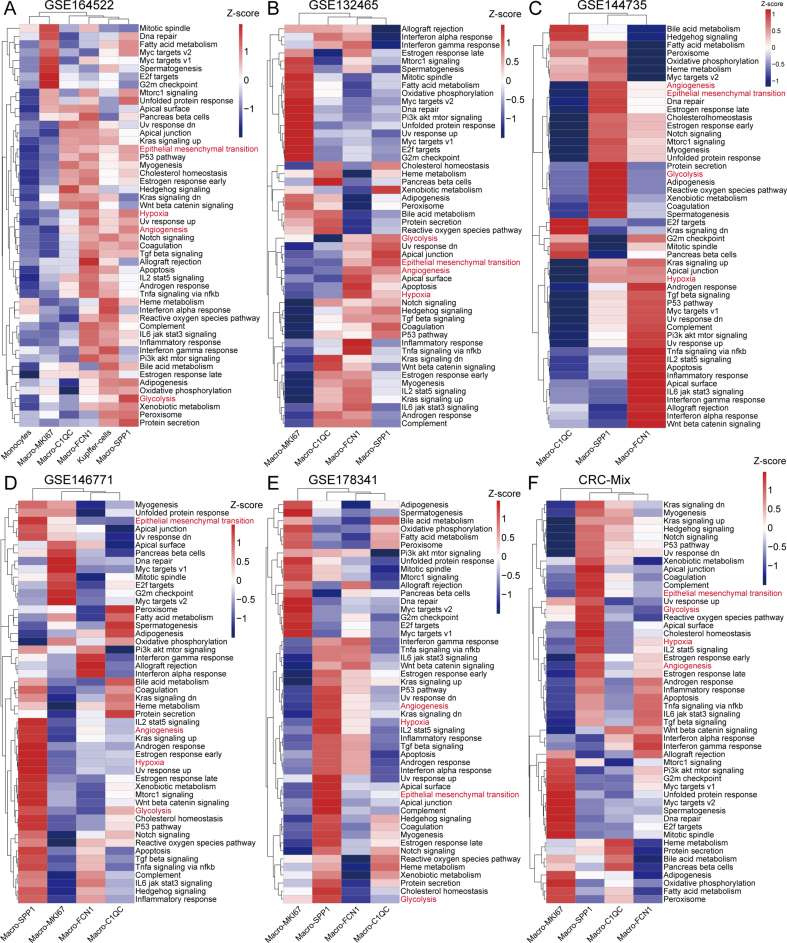
Hallmark gene set enrichment analysis of monocytes/macrophages. **(A)** Heatmap of the GSVA score of 50 hallmark pathways of different monocyte/macrophage subsets (Wu et al). **(B–F)** Heatmap of the GSVA score of 50 hallmark pathways of different macrophage subsets in (B) GSE132465, (C) GSE144735, (D) GSE146771, (E) GSE178341, and (F) CRC-Mix. GSVA, Gene Set Variation Analysis.

We used Monocle2 to observe trajectories of monocyte/macrophage subsets, marker genes, and tissue types during CRLM (Fig. 2G–I). The results showed that monocytes differentiated into *FCN1*^+^ macrophages, which polarized towards *C1QC*^+^ and *SPP1*^+^ macrophages. The expression of SPP1 was elevated in CRC and LM-concentrated regions, suggesting their important role in CRC metastasis. Furthermore, GSVA analysis showed significant enrichment of angiogenesis-, EMT-, glycolysis-, and hypoxia-related genes in *SPP1*^+^ macrophages (Fig. 2J; Fig. S2A–F). This enrichment suggests that *SPP1*^+^ macrophages may be a key subset contributing to the alteration of angiogenesis-, EMT-, and hypoxia-related pathways in monocytes/macrophages during the CRLM process.

### Characteristics and functions of *SPP1*^+^ macrophages in CRC

**Figure 3:**
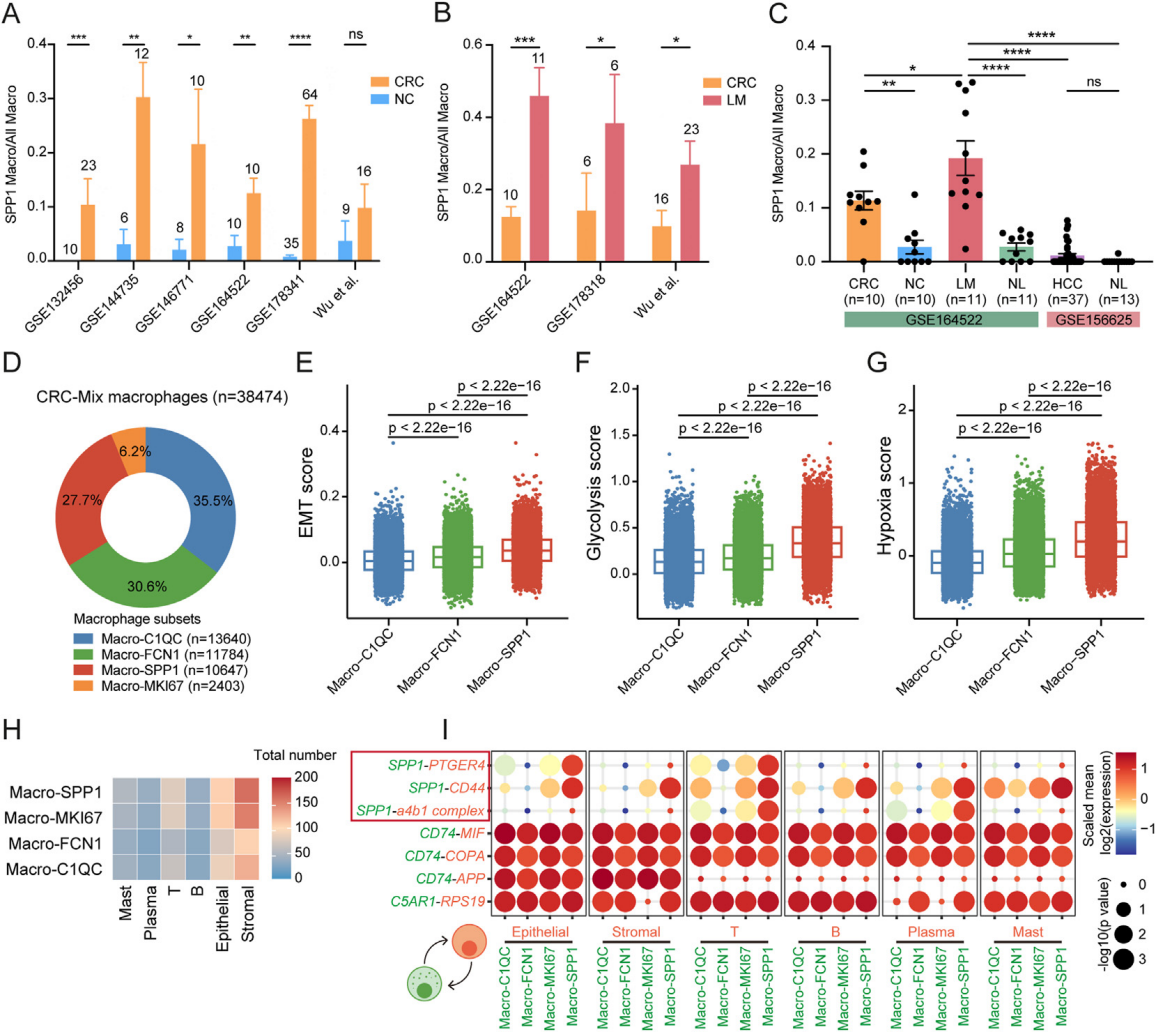
Tissue distribution and potential mechanisms of *SPP1*^+^ macrophages. **(A)** Bar graphs of the ratio of *SPP1*^+^ macrophages to all macrophages in the six scRNA-seq datasets (NC *vs*. CRC). The sample size is shown above the bar. Mean ± standard error of the mean; ∗*P* < 0.05, ∗∗*P* < 0.01, ∗∗∗*P* < 0.001, ∗∗∗∗*P* < 0.0001; ns, not significant; Mann–Whitney U test. **(B)** Bar graphs of the ratio of *SPP1*^+^ macrophages to all macrophages in the three scRNA-seq datasets (CRC *vs*. LM). **(C)** Bar graphs of the ratio of *SPP1*^+^ macrophages to all macrophages in different tissue types (CRC, NC, LM, NL, and HCC) in the GSE164522 and GSE156625 datasets. **(D)** Pie charts of the proportions of four macrophage subsets in the CRC-Mix dataset. **(E–G)** The expression of (E) EMT, (F) glycolysis, and (G) hypoxia signatures in the three major macrophage subsets in the CRC-Mix dataset. **(H)** Heatmap of cellular interactions among the four macrophage subsets and six major cell types (GSE178341). **(I)** Receptor‒ligand interactions among four macrophage subsets and six major cell types (GSE178341). *P* values are indicated by circle size; colors indicate the average expression levels of ligands and receptors or molecules in the corresponding cell types. SPP1, secreted phosphoprotein 1; CRC, colorectal cancer; NC, normal colorectum (adjacent colorectum); LM, liver metastases; NL, normal liver (adjacent); HCC, hepatocellular carcinoma; EMT, epithelial–mesenchymal transition.

Our study found that *SPP1*^+^ macrophages increased in proportion during CRC development and liver metastases, as shown by scRNA-seq data from multiple datasets (Fig. 3A, B and Table S2). This suggests their critical role in CRLM. We also integrated and reclustered CRLM and HCC macrophage data and found that the proportion of *SPP1*^+^ macrophages was significantly lower in HCC than in CRLM samples, indicating that the elevation of *SPP1*^+^ macrophages is cancer-type specific (Fig. 3C and Table S2).

**Figure S3:**
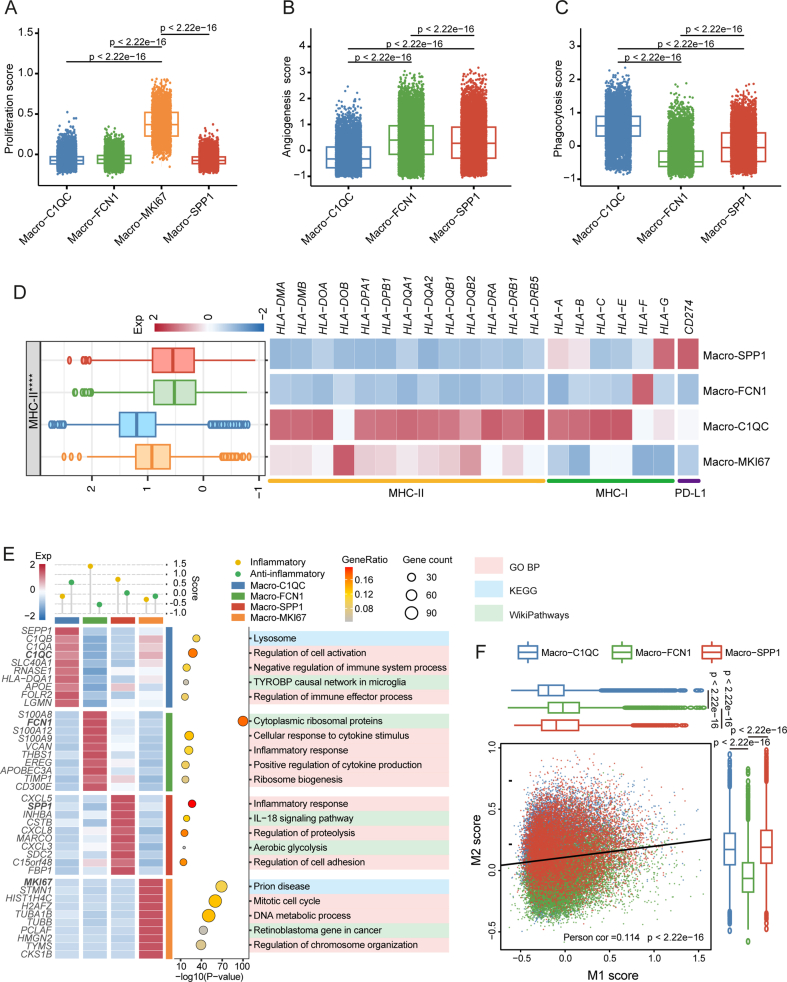
Phenotypes of different macrophage subsets. **(A–C)** The expression of (A) proliferation, (B) angiogenesis, and (C) phagocytosis signatures in different macrophage subsets (CRC-Mix). Mann–Whitney U test. **(D)** Box plots of the expression of the MHC-II signature in the four macrophage subsets (left) and heatmap of the expression of MHC-I molecules, MHC-II molecules, and *PD-L1* in the four macrophage subsets (right). Exp, z score normalized mean expression. **(E)** Heatmap of the mean expression levels of the top 10 significant DEGs in the four macrophage subsets (CRC-Mix) (left). Enrichment analysis of DEGs in each subset of macrophages was performed with Metascape and the top five enriched terms based on GO BP (Gene Ontology Biological Process), KEGG, and WikiPathways gene sets are displayed (right). The lollipop plot shows the expression of inflammatory and anti-inflammatory signatures in the four macrophage subsets (top). **(F)** The upper and right box plots show the expression of M1 and M2 signatures in the three major macrophage subsets, respectively (CRC-Mix). The scatter plot shows the correlation between the M1 and M2 signatures in macrophages. Pearson correlation and linear regression. MHC-I, major histocompatibility complex class I; MHC-II, major histocompatibility complex class II; PD-L1, programmed cell death ligand 1; DEGs, differentially expressed genes.

We analyzed the CRC-Mix dataset to investigate the function of *C1QC*^+^, *FCN1*^+^, *SPP1*^+^, and *MKI67*^+^ macrophages in CRC (Fig. 3D). *MKI67*^+^ macrophages were excluded due to their low proportion and well-defined proliferation function (Fig. S3A). *C1QC*^+^ macrophages were found to have the highest phagocytosis and MHC-II score (Fig. S3B–E), supporting their role in antigen presentation and phagocytosis of pathogens. *FCN1*^+^ macrophages showed higher angiogenesis scores and lower phagocytosis scores than *SPP1*^+^ macrophages (Fig. S3B). *SPP1*^+^ macrophages exhibited specific elevation of EMT, glycolysis, and hypoxia scores, consistent with our GSVA results (Fig. 3E–G).

We assessed the inflammatory, anti-inflammatory, M1, and M2 signatures of different macrophage subsets in CRC-Mix and found that *FCN1*^+^ macrophages had the highest inflammatory score and the lowest anti-inflammatory score, with higher M1 scores and lower M2 scores than *C1QC*^+^ macrophages and *SPP1*^+^ macrophages (Fig. S3E, F). In contrast, *C1QC*^+^ macrophages and *SPP1*^+^ macrophages exhibited more anti-inflammatory features, with *SPP1*^+^ macrophages specifically expressing *CD274* (PD-L1, programmed cell death ligand 1) and *HLA-G* (human leukocyte antigen G),^75^ suggesting an immunosuppressive characteristic (Fig. S3D–F). Additionally, we observed a positive correlation between the M1 signature and the M2 signature in CRC macrophages (Fig. S3F), consistent with findings in multiple tumor scRNA-seq studies.^76^

CellPhoneDB was used to identify ligand‒receptor interactions between *SPP1*^+^ macrophages and other major cell types in CRC. *SPP1*^+^ macrophages interacted with stromal cells, epithelial cells, and T cells through specific ligand‒receptor pairs, including *SPP1*-*CD44*, *SPP1*-*PTGER4*, and *SPP1*-a4b1 complex (Fig. 3H, I). *SPP1* is a core molecule for their biological functions, and *CD44*, *PTGER4* (prostaglandin E receptor 4), and integrin a4b1 are enriched in different cell types.

### SPP1, a novel macrophage marker in CRC

**Figure 4:**
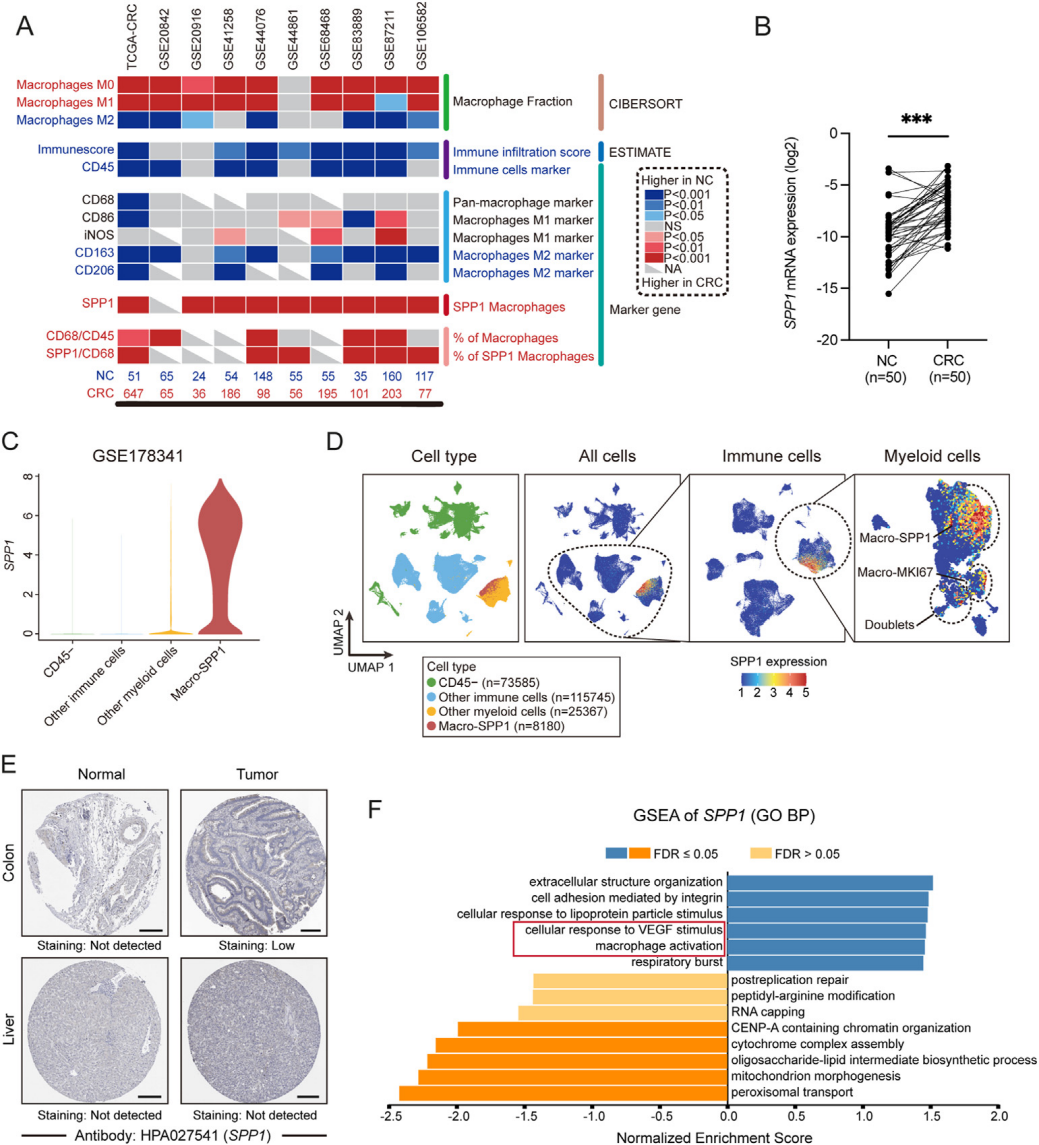
*SPP1* is a novel macrophage marker in CRC. **(A)** Heatmap of the proportion of M0, M1, and M2 macrophages (CIBERSORT), immune score (ESTIMATE), expression of markers (*CD45*, *CD68*, *CD86*, *iNOS*, *CD163*, *CD206*, and *SPP1*), *CD68/CD45*, and *SPP1/CD68* in 10 bulk RNA-seq datasets (NC *vs*. CRC). The color gradient of each tile signifies changes in the marker expression level, where red represents an increase in CRC, blue represents a decrease, and the depth of the color reflects the statistical significance. Additional information provided includes the analysis method (right), dataset source (top), and the sample sizes of NC and CRC for each dataset (bottom). **(B)** Quantitative real-time PCR analysis of *SPP1* mRNA expression between 50 paired NC and CRC samples. Wilcoxon test; ∗∗∗*P* < 0.001. **(C)** Violin plots of the expression of *SPP1* in *CD45*^*−*^ cells, other immune cells, other myeloid cells, and *SPP1*^+^ macrophages (GSE178341). **(D)** UMAP plot of the distribution of *CD45*^*−*^ cells, other immune cells, other myeloid cells, and *SPP1*^+^ macrophages (left). UMAP of iterative subsets of cells from the global level to immune cells to myeloid cells shows the enrichment of *SPP1* expression levels in different cells (right). **(E)** Images of immunohistochemical staining showing *SPP1* protein expression in NC, CRC, NL, and HCC samples. Data are from The Human Protein Atlas. Scale bar, 200 μm. **(F)** Significantly enriched GO BP (Gene Ontology Biological Process) terms in genes coexpressed with *SPP1* in the TCGA-CRC cohort. SPP1, secreted phosphoprotein 1; CRC, colorectal cancer; NC, normal colorectum (adjacent colorectum); NL, normal liver (adjacent); HCC, hepatocellular carcinoma; iNOS, inducible nitric oxide synthase; FDR, false discovery rate.

Bulk RNA-seq datasets were used to investigate changes in macrophage proportions and markers during CRC. CIBERSORT algorithm analysis revealed a significant increase in the proportions of M0 and M1 macrophages and a decrease in M2 macrophages (NC *vs*. CRC) (Fig. 4A). Expression levels of classical macrophage markers showed significant down-regulation of M2 markers *CD163* and *CD206*, while pan-macrophage marker *CD68* and M1 markers *CD68* and *iNOS* remained unchanged.

To reconcile the seemingly inconsistent results on changes in the proportion and number of macrophages during CRC development, we used the ESTIMATE algorithm and *CD45* (*PTPRC*) immune cell marker to assess immune infiltration levels in NC and CRC tissues (Fig. 4A). The results indicated a significant decrease in immune infiltration in CRC tissues, leading to a higher *CD68/CD45* ratio and a significant increase in the proportion of macrophages.

**Figure S4:**
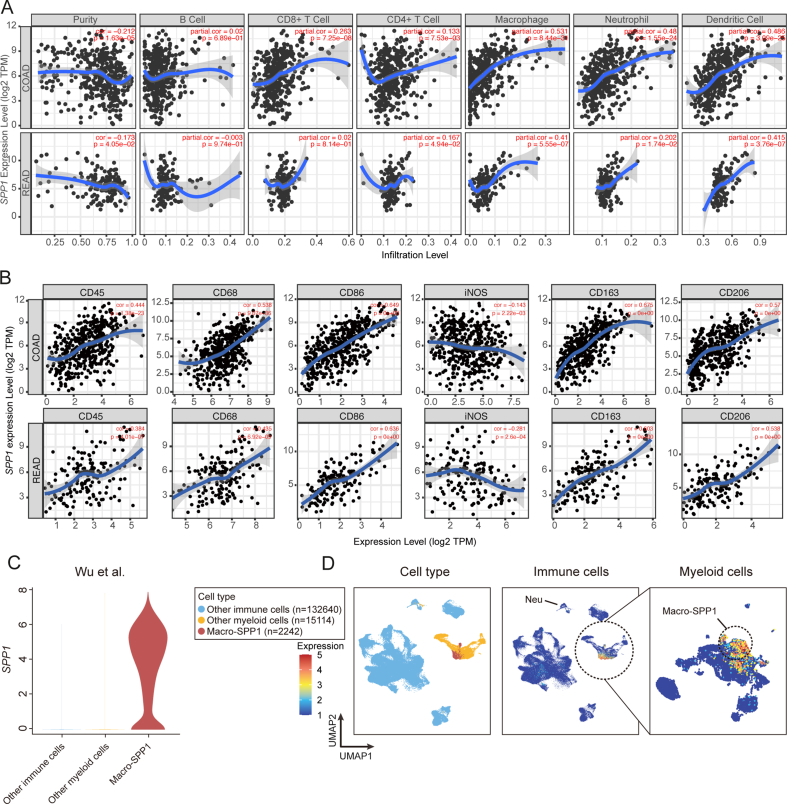
Enrichment of *SPP1* expression in macrophages. **(A)** Correlation analysis between SPP1 expression and immune cells in TCGA COAD/READ (data from TIMER website). Spearman correlation. **(B)** Correlation analysis between SPP1 expression and immune cell markers (*CD45*) and macrophage markers (*CD68*, *CD86*, *iNOS*, *CD163*, and *CD206*) in TCGA COAD/READ (data from the TIMER website). **(C)** Violin plots of the expression of *SPP1* in other immune cells, other myeloid cells, and SPP1^+^ macrophages (Wu et al). **(D)** UMAP plot of the distribution of other immune cells, other myeloid cells, and *SPP1*^+^ macrophages (left). UMAP of iterative subsets of cells from immune cells to myeloid cells shows the enrichment of SPP1 expression levels in different cells (right). SPP1, secreted phosphoprotein 1; iNOS, inducible nitric oxide synthase.

*SPP1* and *SPP1/CD68* were found to be significantly up-regulated in CRC (Fig. 4A). Quantitative real-time PCR analysis of 50 paired samples also confirmed an elevated mRNA expression of *SPP1* in CRC (*P* < 0.001) (Fig. 4B). Correlation analysis indicated a strong correlation between SPP1 expression and the infiltration of macrophages (Fig. S4B), particularly M2 macrophages, in CRC. Furthermore, single-cell sequencing data demonstrated that *SPP1* expression was exclusively enriched in specific myeloid cells in CRC (Fig. 4C, D), indicating that *SPP1* is a specific marker for *SPP1*^+^ macrophages in CRC.

The specificity of *SPP1* as a marker for *SPP1*^+^ macrophages in CRC was further confirmed by immunohistochemistry staining from The Human Protein Atlas (THPA) database,^77^ which showed specific expression of *SPP1* protein in CRC samples but not in NC, NL, and HCC samples (Fig. 4E). GSEA of *SPP1* coexpressed genes in TCGA-CRC using LinkedOmics revealed that extracellular structure organization, cell adhesion mediated by integrin, cellular response to lipoprotein particle stimulus, cellular response to VEGF stimulus, and macrophage activation were among the top significantly enriched GO BP terms (Fig. 4F).

In summary, *SPP1* is a specific biomarker of *SPP1*^+^ macrophages and is significantly elevated in CRC.

### Role of *SPP1*^+^ macrophages in CRC progression, metastasis, and prognosis

**Figure 5:**
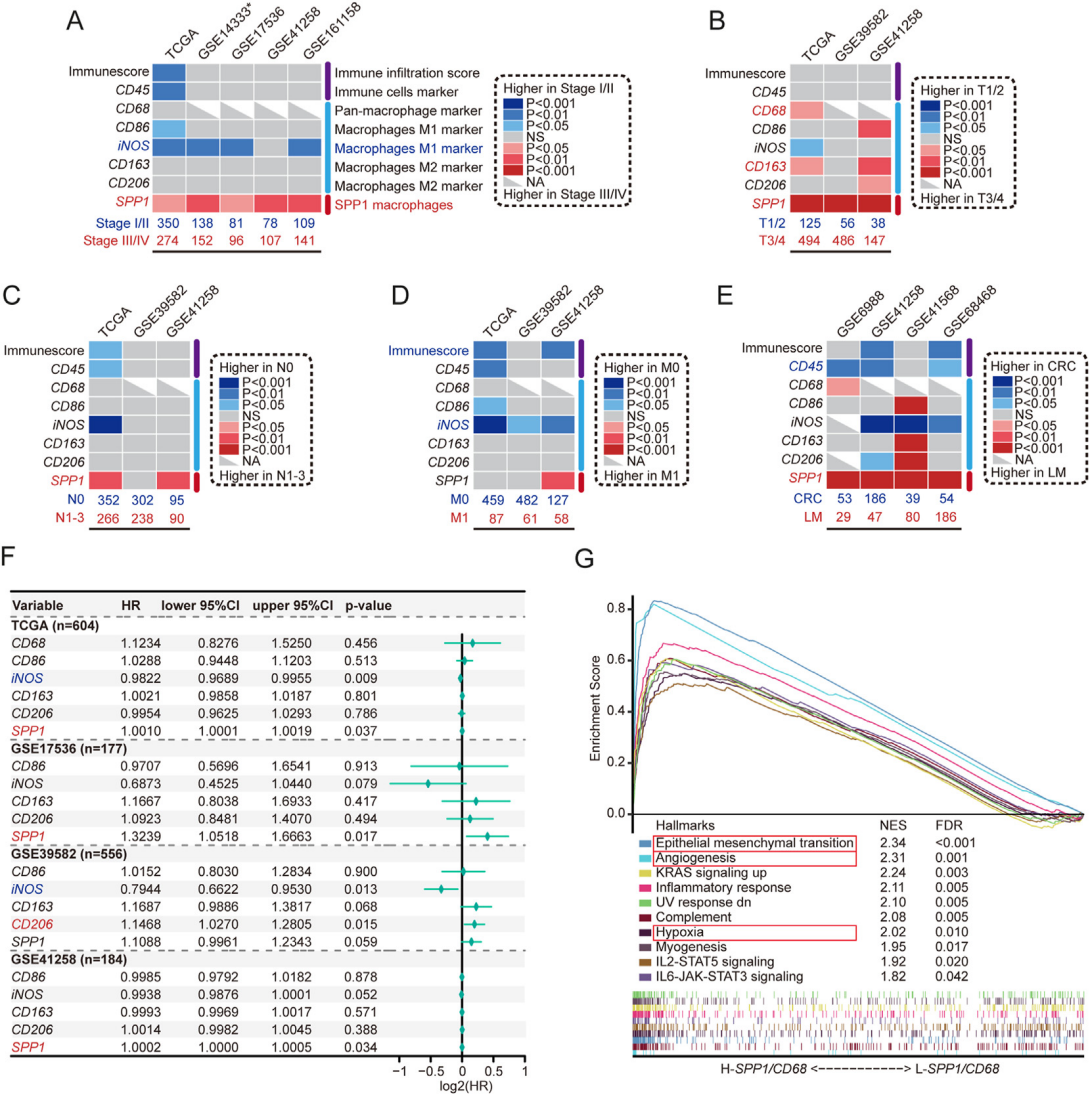
Role of *SPP1*^+^ macrophages in CRC progression, metastasis, and prognosis. **(A–E)** Heatmaps of the immune score (ESTIMATE) and expression of markers (*CD45*, *CD68*, *CD86*, *iNOS*, *CD163*, *CD206*, and *SPP1*) in bulk RNA-seq CRC datasets, comparing stage I/II *vs*. stage III/IV (A), T 1/2 *vs*. T 3/4 (B), N0 *vs*. N1-3 (C), M0 *vs*. M1 (D), and CRC *vs*. LM (E). Top, dataset source; bottom, sample size. ∗GSE14333 uses the Duke stage. **(F)** Univariate Cox analysis of macrophage markers in four CRC datasets (TCGA, GSE17536, GSE39582, and GSE41258). **(G)** The top 10 enriched hallmark terms in the H-*SPP1/CD68* group according to GSEA. SPP1, secreted phosphoprotein 1; CI, confidence interval; GSEA, Gene Set Enrichment Analysis; HR, hazard ratio; iNOS, inducible nitric oxide synthase.

The analysis of bulk CRC datasets showed that *SPP1* expression was elevated in advanced stage (III/IV) CRC (5/5), T3/4 (3/3), and N1-3 (2/3) samples compared with early stage (I/II), T1/2, and N0 samples (Fig. 5A–C), which suggests that *SPP1*^+^ macrophages may play an important role in CRC progression. *SPP1* expression was also significantly higher in LM samples than in CRC samples in four bulk datasets (4/4) (Fig. 5D, E), suggesting the involvement of *SPP1*^+^ macrophages in CRC metastasis.

Macrophage enrichment is generally associated with a poor prognosis in tumors, but in CRC, it is often linked with a favorable prognosis.^12^^,^^78^^,^^79^ Univariate Cox analysis of four datasets (TCGA, GSE17536, GSE39582, and GSE41258) showed that *SPP1* expression was significantly associated with poor overall survival in three of the four datasets (Fig. 5F). Traditional macrophage markers, except for *iNOS* (showing a protective effect), were not significantly associated with CRC progression, metastasis, or prognosis. Considering the colinearity between *SPP1*^+^ macrophages (*SPP1*) and macrophages (*CD68*), *SPP1/CD68* was used to study the relationship between *SPP1*^+^ macrophages and clinical features in CRC (median grouping), revealing that H-*SPP1/CD68* patients had higher mean age, advanced T classification, lymph node metastasis, high levels of microsatellite instability, high tumor mutation burden, and shorter survival time (overall survival and progression-free interval) (Table 2). Furthermore, GSEA revealed the enrichment of hallmark pathways such as EMT, angiogenesis, and hypoxia in the H-*SPP1/CD6*8 group (Fig. 5G).

**Table 2:** Comparison of clinical parameters between the L-*SPP1*/*CD68* and H-*SPP1*/*CD6*8 CRC groups.

Clinical parameters	L-*SPP1*/*CD68* (*n* = 309)	H-*SPP1*/*CD68* (*n* = 308)	*P* value
Age (years)			
Mean ± standard deviation	64.7 ± 12.7	67.8 ± 12.5	0.002^a^
NA	3	0	
Gender			
Female	140 (45.8%)	146 (47.4%)	0.682^b^
Male	166 (54.2%)	162 (52.6%)	
NA	3	0	
Location			
Left	180 (61.2%)	160 (53.2%)	0.047^b^
Right	114 (38.8%)	141 (46.8%)	
NA	15	7	
Pathologic stage			
I	62 (21.3%)	41 (13.5%)	0.078^b^
II	109 (37.5%)	117 (38.6%)	
III	82 (28.2%)	96 (31.7%)	
IV	38 (13.1%)	49 (16.2%)	
NA	18	5	
T classification			
T1	14 (4.6%)	5 (1.6%)	0.005^b^
T2	63 (20.7%)	41 (13.4%)	
T3	199 (65.5%)	219 (71.3%)	
T4	28 (9.2%)	42 (13.7%)	
NA	5	1	
N classification			
N0	182 (60.1%)	165 (53.7%)	0.025^b^
N1	77 (25.4%)	71 (23.1%)	
N2	44 (14.5%)	71 (23.1%)	
NA	6	1	
Metastasis			
M0	216 (85.4%)	236 (82.8%)	0.417^b^
M1	37 (14.6%)	49 (17.2%)	
NA	56	23	
High levels of microsatellite instability			
Yes	32 (10.5%)	50 (16.8%)	0.024^b^
No	272 (89.5%)	247 (83.2%)	
NA	5	11	
Tumor mutation burden			
High	32 (11.7%)	50 (20.5%)	0.006^b^
Low	242 (88.3%)	194 (79.5%)	
NA	35	64	
Overall survival (years)			
Mean (95% confidence interval)	7.97 (6.94–9.01)	6.49 (5.43–7.54)	0.046^c^
Outcome event	55/299	68/294	
Progression-free interval (years)			
Mean (95% confidence interval)	8.09 (7.08–9.09)	6.03 (4.99–7.06)	0.030^c^
Outcome event	67/298	86/293	

Note: The disease location “Left” includes the splenic flexure to the rectum. The disease location “Right” includes the cecum to the transverse colon.

a Mann-Whitney test

b chi-square test

c Log-rank test. High tumor mutation burden, >10 mutations per megabase; CRC, colorectal cancer; SPP1, secreted phosphoprotein 1.

In summary, *SPP1*^+^ macrophages can be used as a biomarker of malignancy to evaluate CRC progression, metastasis, and prognosis.

### Spatial distribution characteristics of *SPP1*^+^ macrophages and their potential impact on the CRC TME

**Figure 6:**
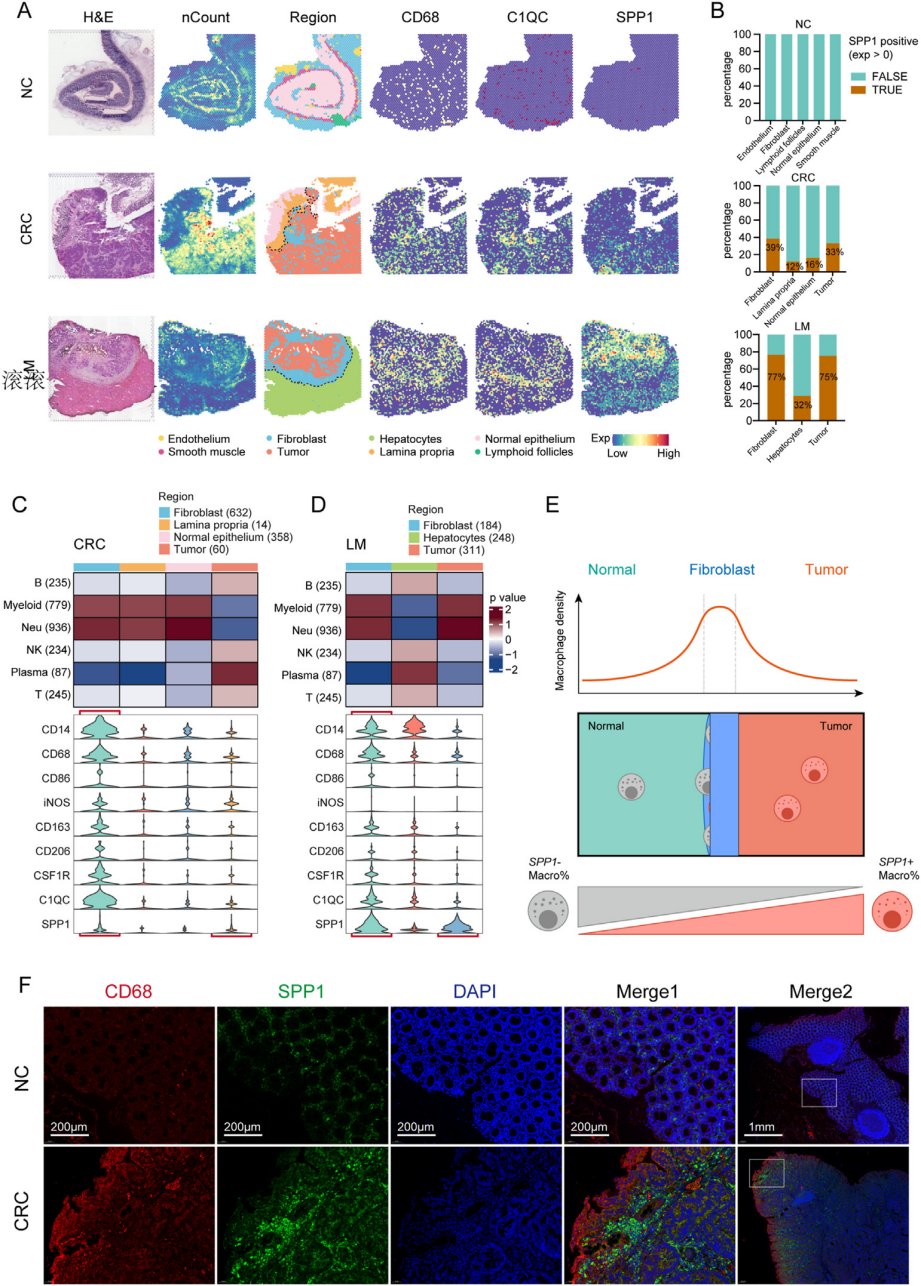
Spatial distribution characteristics of *SPP1*^+^ macrophages and their potential impact on the CRC TME. **(A)** Spatial plots of hematoxylin-eosin staining (column 1), cell count (column 2), region (column 3), and the expression of three macrophage marker genes (column 4–6) in NC (top), CRC (middle), and LM (bottom) samples. **(B)** Stacked plots of the percentage of spots expressing *SPP1* in each region in NC, CRC, and LM samples. **(C)** MIA map of scRNA-seq-identified immune cell types and spatial transcriptomics (ST)-defined regions in CRC (upper). Red indicates enrichment and blue indicates depletion. The violin plot shows the expression of nine macrophage marker genes in ST-defined regions in CRC (lower). **(D)** MIA map of scRNA-seq-identified immune cell types and ST-defined regions in LM samples (upper). The violin plot shows the expression of nine macrophage marker genes in ST-defined regions in LM (lower). **(E)** Schematic diagram of the spatial distribution of macrophages. The fibroblast-enriched area at the tumor border had the highest macrophage density, while the tumor area had the highest proportion of *SPP1*^+^ macrophages. **(F)** Immunofluorescence staining of human CRC tissue and paired NC tissue. *CD68* (red), *SPP1* (green), and DAPI (blue) in individual and merged channels are shown. CRC, colorectal cancer; NC, normal colorectum (adjacent colorectum); LM, liver metastases; MIA, multimodal intersection analysis; SPP1, secreted phosphoprotein 1; TME, tumor microenvironment.

**Figure S5:**
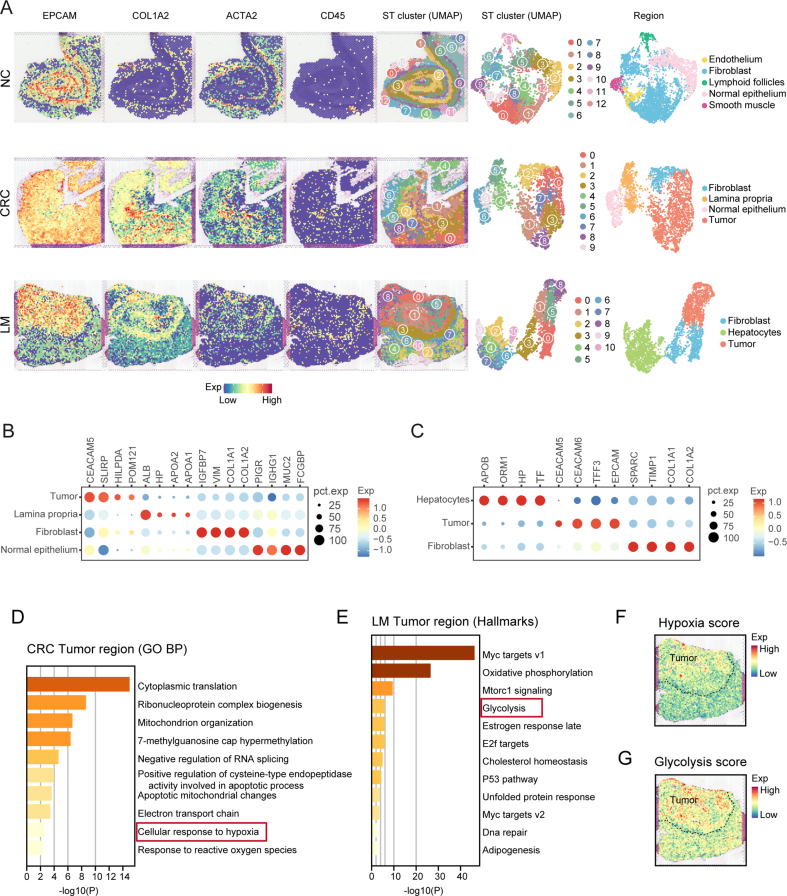
Division of spatial transcriptome-defined regions. **(A)** Spatial plots of the expression of epithelium markers (column 1), fibroblast markers (column 2), smooth muscle markers (column 3), and immune cell markers (column 4) and unbiased clustering of spatial transcriptomics (ST) spots (column 4 and column 5), and ST-defined regions (column 6) in NC (top), CRC (middle), and LM (bottom) samples. **(B)** Dot plots of the average expression of representative genes across different regions in the CRC sample. The dot size represents the percent of spots expressing the genes in each region. The expression intensity of the genes is shown. **(C)** Dot plots of the average expression of representative genes across different regions in the LM sample. **(D)** GO BP (Gene Ontology Biological Process) terms of genes significantly enriched in tumor regions in CRC. **(E)** Hallmark terms of genes significantly enriched in tumor regions in the LM sample. **(F, G)** Spatial feature plots of hypoxia (F) and glycolysis (G) signature scores in the LM sample. CRC, colorectal cancer; NC, normal colorectum (adjacent colorectum); LM, liver metastases.

We performed spatial transcriptomics analysis of NC, CRC, and LM samples to investigate the spatial distribution characteristics of *SPP1*^+^ macrophages (Fig. 6A; Fig. S5A–C). Both CRC and LM samples contained tumor regions, border regions (fibroblast-enriched), and normal tissue regions to ensure representative results. The spots in all regions of the NC samples lacked positive expression of *SPP1*. However, the tumor region and tumor border region (fibroblast region) in CRC and LM samples had significantly higher *SPP1*-positive rates than adjacent normal tissue regions (Fig. 6A, B). The multimodal intersection analysis results of CRC and LM samples showed significant enrichment of myeloid cells in the fibroblast region, and analysis of the expression of nine macrophage markers revealed high macrophage enrichment in the fibroblast region of both CRC and LM samples (Fig. 6C, D). *SPP1* was highly expressed in the tumor regions, unlike other macrophage markers that were only highly expressed in the fibroblast region. These results indicate that although the fibroblast region had the highest macrophage density, the tumor region had the highest proportion of *SPP1*^+^ macrophages (Fig. 6E).

The immunofluorescence results of paired CRC and NC samples confirmed the enrichment of *SPP1*^+^ macrophages in CRC (Fig. 6F). Additionally, these macrophages were found to be distributed closer to the core of the tumor, unlike other macrophages that predominantly concentrated at the boundary region of CRC (Fig. 6F).

We performed enrichment analysis (Metascape) of tumor region-specific genes in CRC and LM samples, which revealed elevated expression of hypoxia- and glycolysis-related pathway genes in tumor regions (Fig. S5D, E). This was further confirmed by the high expression of hypoxia and glycolysis signatures in the tumor region of LM samples (Fig. S5F, G).

In summary, the spatial transcriptomics and immunofluorescence results indicate that *SPP1*^+^ macrophages are enriched in tumor regions, potentially contributing to the hypoxia and glycolysis characteristics of the CRC TME.

## Implications of *SPP1*^+^ macrophages for CRC immunotherapy

**Figure 7:**
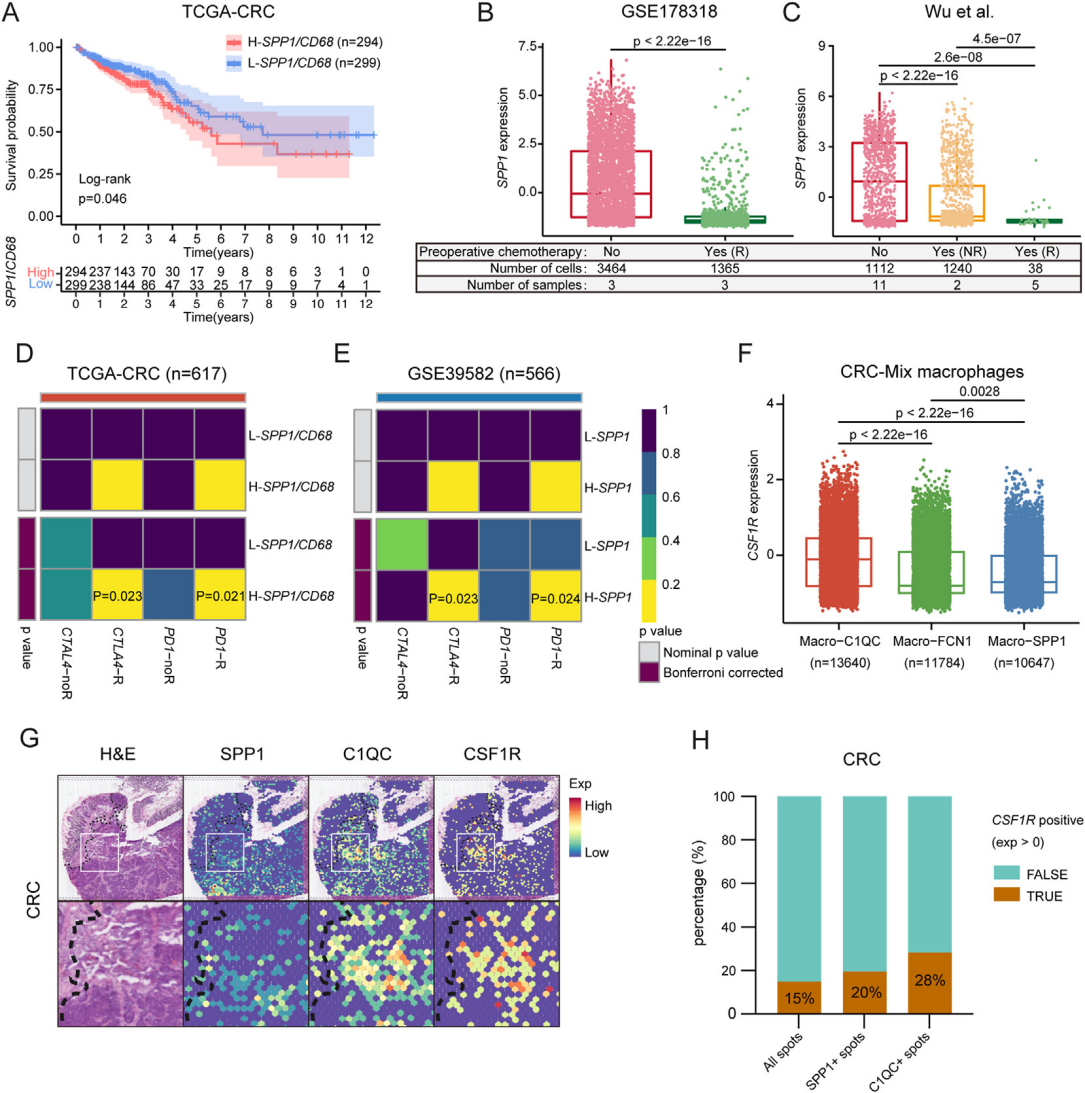
Implications of *SPP1*^+^ macrophages for CRC immunotherapy. **(A)** The Kaplan–Meier survival curve showed the high expression of *SPP1/CD6*8 was associated with worse overall survival (OS) in TCGA-CRC cohorts (*n* = 593). Log-rank test. **(B, C)** Box plots of the effect of preoperative chemotherapy on *SPP1* expression in macrophages in the scRNA-seq cohorts of (B) GSE178318 and (C) Wu et al. R represents responders and NR represents non-responders. Mann–Whitney U test. **(D)** SubMap analysis in TCGA-CRC showed that the H-*SPP1/CD68* group was more sensitive to CTLA4 inhibitors (Bonferroni-corrected *P* = 0.023) and PD-1 inhibitors (Bonferroni-corrected *P* = 0.021) than the L-*SPP1/CD68* group. **(E)** SubMap analysis in GSE39582 showed that the H-*SPP1* group was more sensitive to the CTLA4 inhibitor (Bonferroni-corrected *P* = 0.023) and PD-1 inhibitor (Bonferroni-corrected *P* = 0.024). **(F)** The expression of *CSF1R* in the three major macrophage subsets (CRC-Mix). **(G)** Spatial feature plots of the expression of *SPP1* (column 2), *C1QC* (column 3), and *CSF1R* (column 4) in the CRC sample. **(H)** Stacked plots of the percentage of spots expressing *CSF1R* in regions of all spots (*n* = 3138), *SPP1*^+^ spots, and *C1QC*^+^ spots in the CRC sample. SPP1, secreted phosphoprotein 1; CRC, colorectal cancer; CTLA4, cytotoxic T-lymphocyte associated protein 4; PD-1, programmed death-1; CSF1R, colony-stimulating factor 1 receptor; C1QC, complement C1q C chain.

**Figure S6:**
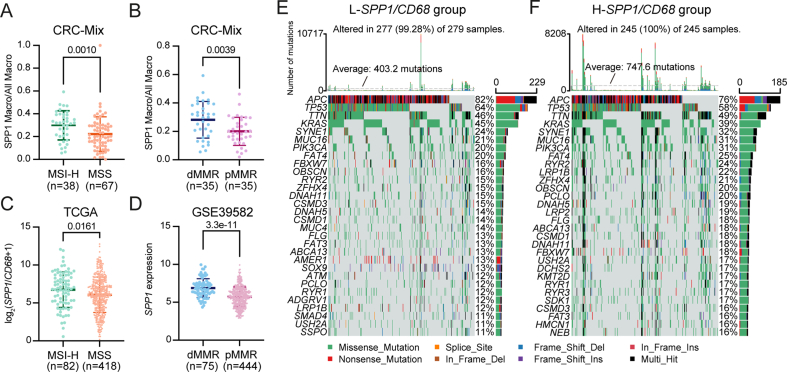
The association between *SPP1*^*+*^ macrophages and genome instability and mutations. **(A)** Comparison of the ratio of *SPP1*^+^ macrophages to all macrophages in MSI-H versus MSS CRC (CRC-Mix). **(B)** Comparison of the ratio of *SPP1*^+^ macrophages to all macrophages in dMMR versus pMMR CRC (CRC-Mix). **(C)** Comparison of *SPP1*/*CD68* in MSI-H versus MSS CRC (TCGA-CRC). **(D)** Comparison of *SPP1* expression in MSI-H versus MSS CRC (GSE39582). **(E)** The waterfall plot shows the mutation profiles of the top 30 mutated genes in the L-*SPP1/CD68* group. Colors of annotations indicate different mutation types (bottom). **(F)** The waterfall plot shows the mutation profiles of the top 30 mutated genes in the H-*SPP1/CD68* group. SPP1, secreted phosphoprotein 1; MSI-H, high levels of microsatellite instability; MSS, microsatellite stability; dMMR, deficient DNA mismatch repair; pMMR, proficient DNA mismatch repair.

**Figure S7:**
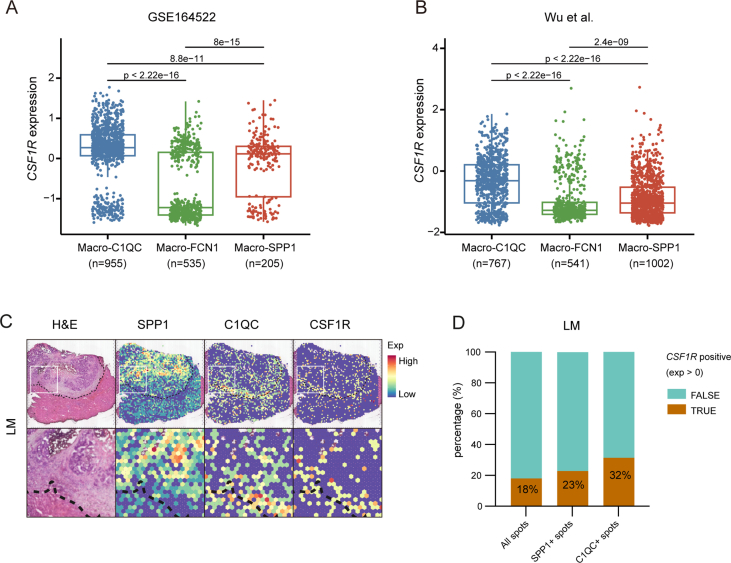
Enrichment of *CSF1R* expression in *C1QC*^+^ macrophages. **(A)** The expression of *CSF1R* in the three major macrophage subsets (GSE164522). **(B)** The expression of *CSF1R* in the three major macrophage subsets (Wu et al). **(C)** Spatial feature plots of the expression of *SPP1* (column 2), *C1QC* (column 3), and *CSF1R* (column 4) in the LM sample. **(D)** Stacked plots of the percentage of spots expressing *CSF1R* in regions of all spots, *SPP1*^+^ spots, and *C1QC*^+^ spots in the LM sample. SPP1, secreted phosphoprotein 1; CSF1R, colony-stimulating factor 1 receptor; C1QC, complement C1q C chain; LM, liver metastases.

Kaplan–Meier analysis of the TCGA-CRC dataset revealed a shorter overall survival in the high *SPP1/CD68* group compared with the low *SPP1/CD68* group (*P* < 0.001) (Fig. 7A). ScRNA-seq data showed that preoperative chemotherapy reduced *SPP1* expression in macrophages (Fig. 7B, C), especially in responders. This suggests that preoperative chemotherapy may reduce *SPP1*^+^ macrophages in CRC TME and may benefit patients. A high proportion of *SPP1*^+^ macrophages was associated with genome instability and mutation (DNA mismatch repair deficiency, high levels of microsatellite instability, and high tumor mutation burden) in CRC (Fig. S6A–F), indicating that they could benefit from immunotherapy. SubMap algorithms predicted that the H-*SPP1/CD68* group was more likely to respond to anti-PD-1 therapy and anti-CTLA-4 therapy (Fig. 7D, E). *CSF1R* expression was significantly enriched in *C1QC*^+^ macrophages but not *SPP1*^+^ macrophages (Fig. 7F–H; Fig. S7A–D), suggesting that blocking *CSF1R* would preferentially deplete *C1QC*^+^ macrophages over *SPP1*^+^ macrophages.

## Discussion

**Figure 8:**
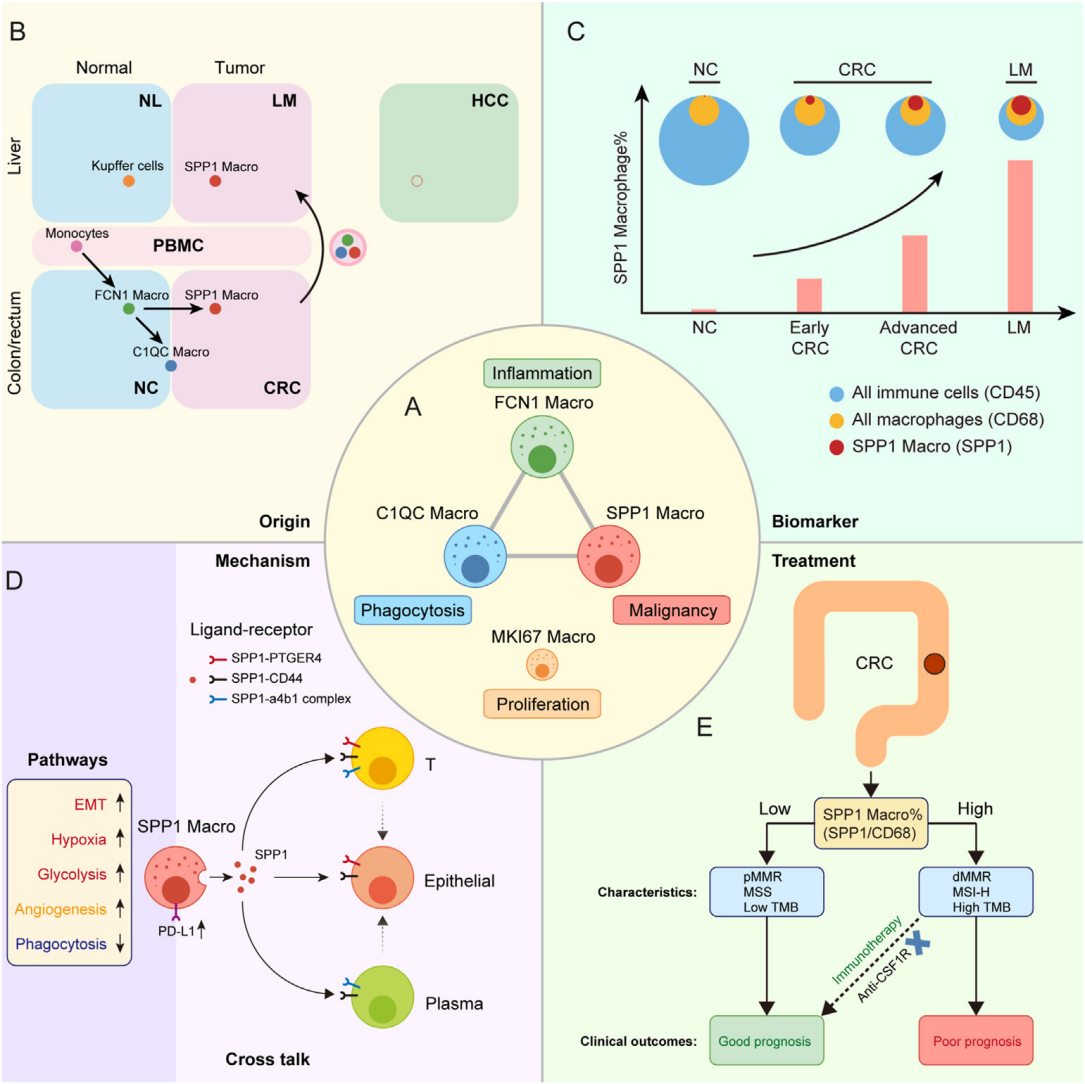
Schematic diagram of the *SPP1*^+^ macrophage model in CRC. **(A)** Classification of macrophage subsets in CRC, including *FCN1*^+^, *C1QC*^+^, *SPP1*^+^, and *MKI67*^+^ macrophages, defined by their core features of inflammation, phagocytosis, malignancy, and proliferation, respectively. **(B)** Model of the developmental trajectory of monocyte/macrophage lineages in different sample types from CRLM and HCC patients. This model shows the cell origin and tissue distribution of *SPP1*^+^ macrophages. **(C)** The number and proportion of *SPP1*^+^ macrophages increased during CRC occurrence, progression, and metastasis, making them a marker of CRC malignancy. Blue, yellow, and red dots represent the numbers of all immune cells, all macrophages, and *SPP1*^+^ macrophages in the sample, respectively. **(D)***SPP1*^+^ macrophages had distinct signatures compared with other macrophages (left). Red: signature specifically elevated in *SPP1*^+^ macrophages. Yellow: signature elevated in *SPP1*^+^ macrophages compared with *C1QC*^+^ macrophages. Blue: signature decreased in *SPP1*^+^ macrophages compared with *C1QC*^+^ macrophages. The right shows the crosstalk between *SPP1*^+^ macrophages and other cell subsets through the *SPP1* protein. **(E)** Patients with a high proportion of *SPP1*^+^ macrophages have a worse prognosis, along with signatures related to genome instability and mutations. Immunotherapy has the potential to improve outcomes in patients with a high proportion of *SPP1*^+^ macrophages, whereas targeting *CSF1R* is less effective. SPP1, secreted phosphoprotein 1; CSF1R, colony-stimulating factor 1 receptor; CRC, colorectal cancer; CRLM, colorectal cancer liver metastases; HCC, hepatocellular carcinoma; C1QC, complement C1q C chain.

This study provides a comprehensive analysis of *SPP1*^+^ macrophages in CRC, including their origin, distribution, clinical value, functional pathways, and implications for treatment (Fig. 8A–E). Based on the results, the authors propose the *SPP1*^+^ macrophage model theory, which explains the changes in macrophages during CRLM and guides clinical diagnosis and treatment.

Four macrophage subsets were identified from CRC tissue, including *FCN1*^+^ macrophages, *C1QC*^+^ macrophages, *SPP1*^+^ macrophages, and *MKI67*^+^ macrophages (Fig. 8A). Enrichment analysis and phenotypic signatures indicated that inflammation, phagocytosis, malignancy, and proliferation were the most prominent features related to these four macrophage subsets, respectively.

The *SPP1*^+^ macrophages were specifically enriched in primary foci and liver metastases of CRC, and evidence from trajectory and tissue distribution analysis supports the hypothesis that they originate from monocytes recruited into colonic tissues and rapidly differentiate into macrophages, which then differentiate into monocyte-like *FCN1*^+^ macrophages that polarize toward *C1QC*^+^ macrophages and *SPP1*^+^ macrophages (Fig. 8B).

Myeloid cells play an active role in shaping the CRC TME,^35^ with a significant increase in the proportion of myeloid cells and macrophages confirmed in our study. Interestingly, the number of macrophages in tissues did not change significantly during CRC tumorigenesis, but the decrease in overall immune cells resulted in a significant increase in the proportion of macrophages (Fig. 8C). This suggests that anti-tumor immunity weakens as CRC progresses, while the importance of macrophages in the TME increases.

*SPP1*^+^ tumor-associated macrophages have been associated with CRC malignancy and liver metastasis,^19^^,^^20^^,^^32^^,^^33^ and *SPP1* is a diagnostic and prognostic marker for CRC.^22^^,^^28^^,^^80^^,^^81^ Our study confirms *SPP1*^+^ macrophages as a comprehensive marker for CRC diagnosis, disease severity assessment, and prognosis evaluation, with their number and proportion constantly increasing during CRC occurrence, progression, and metastasis, indicating a poor prognosis (Fig. 8C). These results suggest that *SPP1*^+^ macrophages play an important driving role in the occurrence, progression, and metastasis of CRC.

The *in vitro* M1/M2 polarization model has limitations in describing macrophages in the complex phenotype of the TME.^8^ Coexpression of M1 and M2 signatures in macrophage subsets in cancer, including CRC, has been reported by single-cell studies.^19^^,^^32^^,^^57^^,^^76^ In our study, we also found an increase in the proportion of M1 macrophages and a decrease in M2 macrophages in CRC, contradicting the M1/M2 polarization theory. The biological function of *SPP1*^+^ macrophages in CRC remains unclear, but our study suggests they may promote CRC through EMT, hypoxia, glycolysis, and immunosuppressive pathways (Fig. 8D). *SPP1* is a key functional molecule of *SPP1*^+^ macrophages, and these macrophages interact with other cells through the *SPP1-CD44*, *SPP1-PTGER4*, and *SPP1*-a4b1 complex axes. This suggests that *SPP1*^+^ macrophages may promote CRC progression and metastasis through these interactions (Fig. 8D).

The understanding of *SPP1*^+^ macrophages presents new potential for CRC treatment. Chemotherapy-induced macrophage phenotype switching has been reported in breast cancer,^82^ and our findings suggest that preoperative chemotherapy can significantly reduce *SPP1* expression in CRC macrophages, especially in responders. This suggests that effective chemotherapy may inhibit *SPP1*^+^ macrophages specifically and raise the possibility of immunotherapy targeting *SPP1*^+^ macrophages. Importantly, patients with high *SPP1*^+^ macrophage levels show higher levels of genomic instability and mutation characteristics that predict better responses to immune checkpoint blockade (Fig. 8E). Our findings are supported by the enrichment of *PD-L1* and *HLA-G* in *SPP1*^+^ macrophages and prediction results of the SubMap analysis. Several studies have shown that targeted blockade of *SPP1* can inhibit tumor progression and metastasis.^21^^,^^25^^,^^83^, ^84^, ^85^ Thus, targeting *SPP1*^+^ macrophages may offer a viable option for CRC immunotherapy.

Notably, our *SPP1*^+^ macrophage theory also reveals a possible problem in current macrophage-targeted immunotherapy research. Inhibition of the *CSF1-CSF1R* axis to reduce tumor-associated macrophages is a major research direction of macrophage-targeted immunotherapy.^6^^,^^13^, ^14^, ^15^ However, we found that *CSF1R* was significantly enriched in *C1QC*^+^ macrophages compared with other macrophages, suggesting that *CSF1R* blockade may preferentially deplete the protective *C1QC*^+^ macrophage subset while sparing malignant *SPP1*^+^ macrophages. Anti-*CSF1R* therapy may not be sufficient to deplete all tumor-promoting *SPP1*^+^ macrophages, which may contribute to the poor efficacy of anti-*CSF1R* monotherapy in the Renca mouse tumor model and human cancer patients.^15^^,^^16^^,^^19^

In summary, we propose an *SPP1*^+^ macrophage model to explain macrophage characteristics and changes in CRC. *SPP1*^+^ macrophages are a marker of malignancy and targeting them would serve as a promising CRC therapeutic strategy.

## Ethics declaration

The research was approved by the Medical Ethics Committee of the First Affiliated Hospital of the Fourth Military Medical University (Approval No.: KY20212211–C-1). The patients/participants provided their written informed consent to participate in this study. Written informed consent was obtained from the individual(s) for the publication of any potentially identifiable images or data included in this article.

## CRediT authorship contribution statement

Z.Y.X. designed the study. Z.Y.X. and L.R.N. collected the data. Z.Y.X., F.F., and G.Z.Z. analyzed the data. Z.Y.X., K.L.D., R.K.L., H.J.D., L.L.D., H.Z.W., G.M.R., and X.Y.D. visualized the data. Z.Y.X. and L.R.N. drafted the manuscript. Z.Y.X., G.Z.Z., J.Z., S.C.D., and J.Y.Z. revised the manuscript. All authors read and approved the final manuscript. All authors contributed to the manuscript and approved the submitted version.

## Conflict of interests

The authors declared no conflict of interests.

## Funding

This work was supported by the National Natural Science Foundation of China (No. 82072655), the Scientific and technological innovation team of Shaanxi Innovation Capability Support Plan (China) (No. 2023-CX-TD-67), and the Key R&D Plan of Shaanxi Province, China (No. 2022SF-603).

## Data availability

We utilized publicly available datasets in this study, and their details can be found in the “Methods” section of the manuscript. The codes used in this study are accessible on GitHub (https://github.com/LPC19970117/SPP1-macrophages). Further inquiries can be directed to the corresponding authors.

## Ethic approval

Human tissue specimens were obtained from Xijing Hospital under the approval of the Institutional Review Board and have obtained informed consent from the patients.

Review approval document of the medical ethics committee of the first affiliated hospital of the air force medical university (Xijing Hospital).

Approval number: KY20212211–C-1.

## Appendix A Supplementary data

Table S1 Signature gene lists. Table S1

Table S2 Major cell type composition, myeloid cell subset composition, and clinical information for each sample. Table S2

Table S3 Differentially expressed genes in myeloid subgroups in CRC-Mix. Table S3

Table S4 Differentially expressed genes in monocytes/macrophages between normal colorectum and colorectal cancer. Table S4

Table S5 Differentially expressed genes in monocytes/macrophages between colorectal cancer and liver metastases. Table S5
